# Expression of the Carboxy-Terminal Portion of MUC16/CA125 Induces Transformation and Tumor Invasion

**DOI:** 10.1371/journal.pone.0126633

**Published:** 2015-05-12

**Authors:** Thapi D. Rao, Huasong Tian, Xun Ma, Xiujun Yan, Sahityasri Thapi, Nikolaus Schultz, Nestor Rosales, Sebastien Monette, Amy Wang, David M. Hyman, Douglas A. Levine, David Solit, David R. Spriggs

**Affiliations:** 1 Department of Medicine, Memorial Sloan Kettering Cancer Center; and Department of Medicine, Weill Cornell Medical College, New York, NY, United States of America; 2 Computational Biology Center, Memorial Sloan Kettering Cancer Center, New York, NY, United States of America; 3 Tri-Institutional Laboratory of Comparative Pathology, Memorial Sloan Kettering Cancer Center, The Rockefeller University, Weill Cornell Medical College, New York, NY, United States of America; 4 Department of Medicine, Memorial Sloan Kettering Cancer Center; Weill Cornell Medical College, New York, NY, United States of America; 5 Gynecology Service, Department of Surgery, Memorial Sloan Kettering Cancer Center; and Department of Obstetrics and Gynecology, Weill Cornell Medical College, New York, NY, United States of America; Sun Yat-sen University Medical School, CHINA

## Abstract

The CA125 antigen is found in the serum of many patients with serous ovarian cancer and has been widely used as a disease marker. CA125 has been shown to be an independent factor for clinical outcome in this disease. In The Cancer Genome Atlas ovarian cancer project, MUC16 expression levels are frequently increased, and the highest levels of MUC16 expression are linked to a significantly worse survival. To examine the biologic effect of the proximal portion of MUC16/CA125, NIH/3T3 (3T3) fibroblast cell lines were stably transfected with the carboxy elements of MUC16. As few as 114 amino acids from the carboxy-terminal portion of MUC16 were sufficient to increase soft agar growth, promote matrigel invasion, and increase the rate of tumor growth in athymic nude mice. Transformation with carboxy elements of MUC16 was associated with activation of the AKT and ERK pathways. MUC16 transformation was associated with up-regulation of a number of metastases and invasion gene transcripts, including IL-1β, MMP2, and MMP9. All observed oncogenic changes were exclusively dependent on the extracellular “ectodomain” of MUC16. The biologic impact of MUC16 was also explored through the creation of a transgenic mouse model expressing 354 amino acids of the carboxy-terminal portion of MUC16 (MUC16^c354^). Under a CMV, early enhancer plus chicken β actin promoter (CAG) MUC16^c354^ was well expressed in many organs, including the brain, colon, heart, kidney, liver, lung, ovary, and spleen. MUC16^c354^ transgenic animals appear to be viable, fertile, and have a normal lifespan. However, when crossed with p53-deficient mice, the MUC16^c354^:p53^+/-^ progeny displayed a higher frequency of spontaneous tumor development compared to p53^+/-^ mice alone. We conclude that the carboxy-terminal portion of the MUC16/CA125 protein is oncogenic in NIH/3T3 cells, increases invasive tumor properties, activates the AKT and ERK pathways, and contributes to the biologic properties of ovarian cancer.

## Introduction

**Fig 1:**
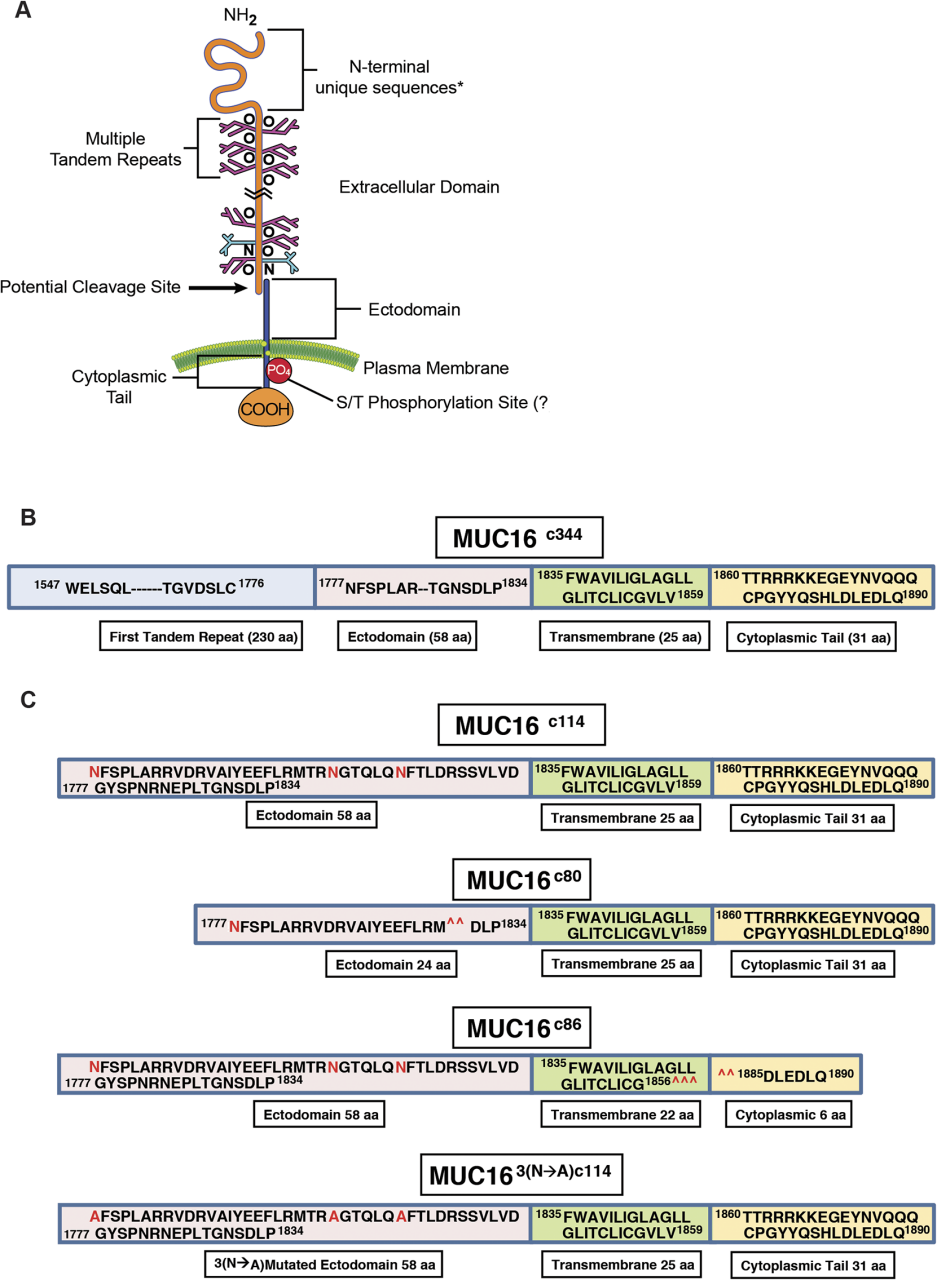
Features of MUC16. A) Schematic illustration of all MUC16 elements; B) Linear representation of the truncated MUC16^c344^ construct; C) Linear representation of the truncated MUC16^c114^, constructs with 3 N-glycosylation sites in red, two deletion mutants MUC16^c80^ and MUC16^c86^, removing either external or internal portions of the MUC16 molecule and mutated 3 asparagine N-glycosylation sites to alanine (MUC16^3(N—A)c114^), as detailed in S1 Fig.

The serum CA125 antigen has been a mainstay of ovarian cancer assessment and management since the early 1980’s, but its biology and contribution to ovarian cancer manifestations have been poorly understood [1–3]. The cloning of CA125, achieved in 2001, first identified MUC16 as a tethered mucin with a small intracellular domain, a transmembrane domain, an ectodomain proximal to the putative cleavage site, and a large, heavily glycosylated region of 12–20 tandem repeats, each 156 amino acids long (Fig 1A) [4–6]. Serous cancers of the ovary, fallopian tube, and uterus often express large amounts of MUC16, and aberrant MUC16 expression can be found in several other malignancies, including cancers of the lung, pancreas, and breast. Expression of other tethered mucins is a common feature of epithelial organs, and they are often over-expressed in malignancy. Two prominent examples are MUC1, which is over-expressed in many breast and ovarian cancers, and MUC4, which is characteristically abundant in pancreatic and gastrointestinal cancers [7]. Both of these mucins have been identified as having transforming properties [8, 9]. The transforming mechanisms are different and incompletely understood. MUC1 has a β-catenin homology region that has been shown to translocate to the nucleus and act as a transcription factor. In contrast, MUC4 has HER-binding domains within its transmembrane region and acts, at least in part, through the HER family kinases [10, 11]. MUC16 lacks homologous regions to either of these domains and appears to have evolved independently [12]. Compared to both MUC1 and MUC4, the expression of MUC16 is more restricted and is normally expressed, almost exclusively, to the Müllerian tract and the ocular epithelium [13–15]. The tandem repeat regions of the MUC16 molecule appear to function as key interacting proteins with mesothelin and other stromal proteins [16]. These interactions are probably responsible for the classic patterns of serosal spread by ovarian cancers. Others have identified the importance of the carboxy-terminal portion in invasion and growth, but the specific regions of the proximal muc16 sequence responsible for transformation have not been delineated [17, 18]. In clinical settings, high levels of the circulating elements from MUC16, which encode the CA125 antigen, are associated with an adverse clinical outcome, independent of stage, grade, and other traditional clinical factors [19]. Amplification of genomic regions encoding MUC16 in ovarian cancer DNA and over-expression of MUC16 mRNA have been observed in The Cancer Genome Atlas (TCGA) ovarian cancer project and is associated with worse outcome [20]. Loss of MUC16 in the mouse is not associated with a distinct phenotype, but the effect of persistent or aberrant MUC16 expression is not known [21]. We hypothesized MUC16 is important in the serous ovarian cancer phenotype and suspected that expression of key elements from the MUC16 protein could promote invasive behavior in preclinical models of cancer, both *in vitro* and *in vivo*. Our experimental results strongly indicate that the expression of the most proximal MUC16 fragments is associated with specific alterations of signal transduction, gene expression, and aggressive biological behavior.

## Results

Following apparent cleavage and release of the tandem repeat region, approximately 114 amino acids of the carboxy-terminal portion of the protein are thought to remain on the cell surface, and the potential functions of this part of the molecule are not known. We examined the role of this most proximal part of the MUC16 protein in malignant transformation and behavior in 3T3 fibroblasts and ovarian cancer cell lines. To test the effect of the residual c114 amino acid element proximal to the cleavage site, we designed two vectors: 1) MUC16^c114^-GFP vector and 2) the truncated MUC16^c344^-GFP vector; and we transfected both of these vectors, along with the phrGFP control vector, into 3T3 fibroblast cells (Fig 1B and 1C and S1A and S1B Fig). MUC16-expressing cell lines were selected and maintained with G418, and MUC16 stable expression was confirmed by fluorescence activated cell sorting (FACS) analysis using both green fluorescent protein (GFP) detection and monoclonal antibodies that recognize unique amino acid sequences of the MUC16 carboxy-terminal portion [13]. The cell lines that express c344 amino acids from the MUC16 protein (MUC16^c344^-GFP lines) bear the classic CA125 epitope, recognized by the OC125 antibody, on the cell surface by FACS analysis and elaborated into the cell culture supernatant (data not shown). However, all of the transfected lines were cell surface positive for the MUC16^c114^ extracellular sequences, proximal to the putative cleavage site and recognized by the MUC16 ectodomain-specific 4H11 antibody, with similar amounts of MUC16 present by FACS analysis [13] (S2 Fig).

### 3T3 Cells

**Fig 2:**
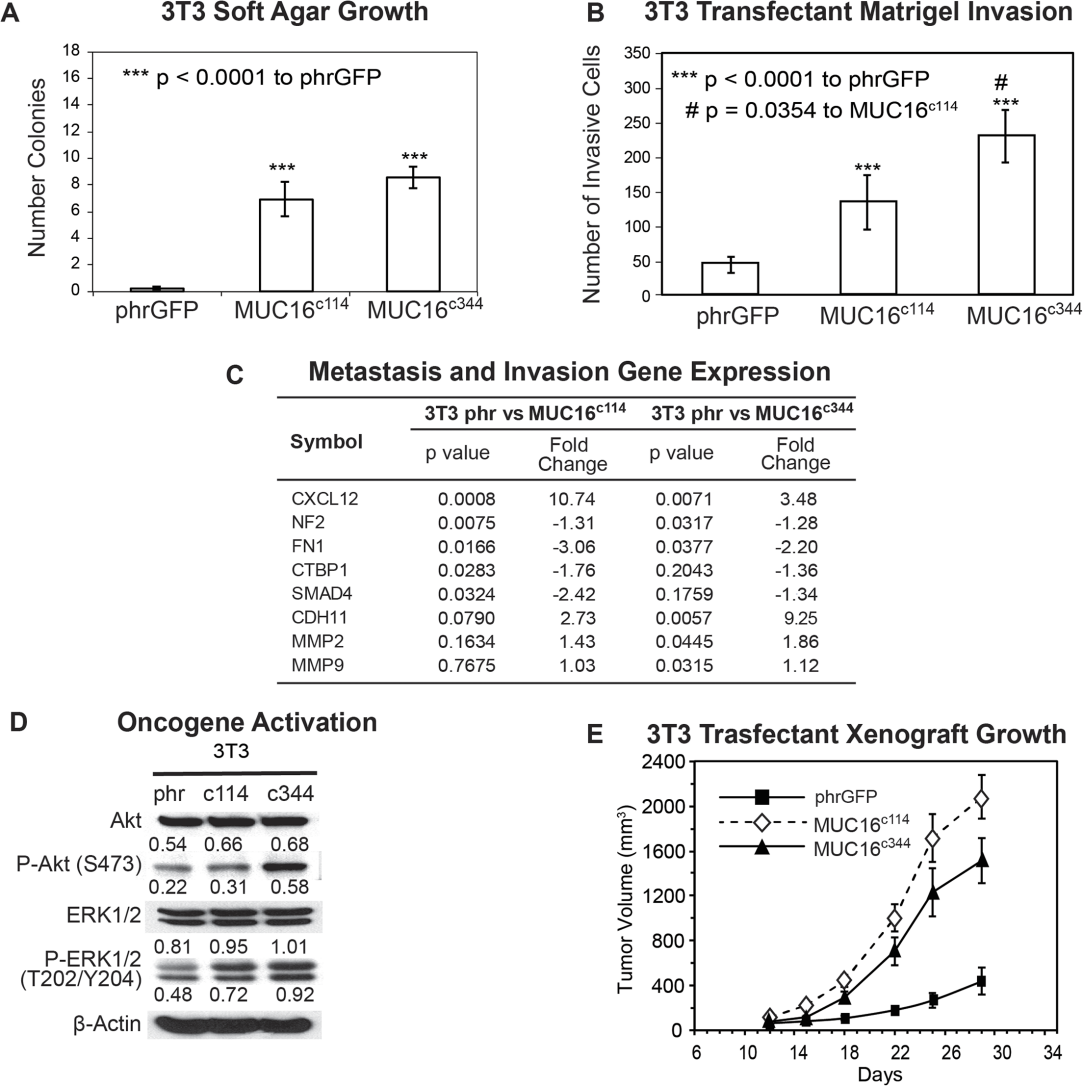
Effect of MUC16 in 3T3 cells. A) Soft agar growth of 3T3 transfectants in 60 mm dishes. After 14 days, colonies were counted and plotted. The data shown in the table represent one of three similar experiments (*** p < 0.0001) compared to the phrGFP control vector; B) Matrigel invasion assay for 3T3 cell lines following stable transfections with either the phrGFP control vector, MUC16^c114^-phrGFP or MUC16^c344^ phrGFP carboxy-terminal constructs. Each assay was performed two or more times in triplicate and counted by hand. Both MUC16^c114^ 3T3 and MUC16^c344^ 3T3 cell lines were significantly more invasive (*** p<0.0001) compared to the phrGFP 3T3 vector control, and the MUC16^c344^ cell line is significantly more invasive than the MUC16^c114^ cell line (# p = 0.0354). C) Expression of metastasis and invasion genes induced by MUC16^c114^ and MUC16^c344^ expression. A SuperArray panel of 80 invasion/metastasis gene transcripts was examined for MUC16-positive and vector only cell lines. The expression of selected chemotactic, adhesion, and invasion transcripts was measured in 3T3 MUC16^c114^ or 3T3-MUC16^c344^ cell lines (each of three triplicates was examined in duplicate and compared to the phr vector only controls by chi square testing). The p value for each transcript, adjusted for repeated measures, is shown in the table. All genes with changes at the corrected p<0.05 or below level are included. D) Transfected 3T3 cells were examined for activation of the ERK/AKT signaling pathways compared to the vector only controls. Phosphorylation of ERK1/2 (pT202/Y204) and AKT (S473) was increased following MUC16^c114^ and MUC16^c344^ constructs, compared to the phrGFP vector. Activation of both pathways was seen in each of the cell lines. β-Actin normalized densitometry quantification values are shown below each Western blot band. E) MUC16 transfectant tumor growth in athymic nude mice. Two million tumor cells were introduced into the flank of 15 nu/nu mice, and the mice were observed for tumor formation. Tumors were measured by calipers twice weekly. The differences in mean tumor volume were significantly greater for mice bearing MUC16-positive tumors (both lines p<0.0001 compared to the phrGFP control vector).

To first investigate the transforming properties conferred by the residual, post cleavage elements of MUC16, we examined the characteristics of the 3T3 MUC16^c114^-GFP and 3T3 MUC16^c344^-GFP cell lines and compared the effects of these two minimal MUC16 elements to the vector controls. Expression of either the most proximal 114 amino acids or the proximal 344 amino acids of the MUC16 sequence had no significant effect on the *in vitro* growth rates for any of the transfected cell lines when compared with that of the parental line (S3 Fig). However, expression of the same elements of the MUC16 protein substantially altered 3T3 anchorage dependent growth in soft agar cloning. Both the minimal c114 and the longer c344 MUC16 fragments significantly increased the number of soft agar colonies compared to the vector only controls (Fig 2A). The MUC16^c344^ 3T3 transfectants were similarly proficient in soft agar colony formation compared to the MUC16^c114^ cells. Both the c114 and c344 proximal portions of MUC16 protein expression also enhanced the migration (p<0.0001) of MUC16-positive 3T3 cells in classic matrigel invasion assays compared to the 3T3 cells transfected with phrGFP vector controls (Fig 2B). However, compared to the MUC16^c114^ cells, the MUC16^c344^ were even more invasive in the matrigel assay. When the 3T3 cells expressing various MUC16 protein fragments were examined for expression of selected metastasis and invasion gene transcripts, there were multiple invasion genes upregulated, including chemokine ligand 12 (CXCL12), Cadherin 11 (CDH11), and the matrix metalloproteinases MMP2 and MMP9 (Fig 2C). Other transcripts including Fibronectin (FN1) and Neurofibromin (NF2) are consistently decreased. It is notable that the MUC16^c114^ and MUC16^c344^ transfectants had many of the same directional changes in the invasion/adhesion transcripts compared to the vector only controls. However, the MUC16c^344^ showed higher expression of MMP2 and MMP9, which did not reach statistical significance in the MUC16^c114^ cells, possibly reflecting additional contribution from the additional elements. Since carboxy elements of MUC16 increase invasive properties of cells bearing the MUC16 protein, we hypothesized that MUC16 might act through canonical signaling pathways in ways similar to the effects of MUC1 and MUC4. The interacting ERK and AKT pathways have previously been identified as important signaling mechanisms in ovarian cancer and regulators of tumor cell invasion [22–24]. As shown in Fig 2D, there was activation of both pathways as evidenced by increases in pAKT(S473) and pERK (T202/Y204). There were modest changes in both ERK and AKT transcripts, which were much more remarkable in the phosphorylated species (pERK and pAKT). The pathways in the MUC16^c344^ 3T3 cells were even more activated than in the MUC16^c114^ 3T3 cells.

The most unambiguous hallmark of oncogenic transformation is the ability to promote growth in immunodeficient mice. In order to measure the effects of MUC16 on tumor growth rate, we selected a flank tumor model to facilitate regular tumor measurements. As shown in Fig 2E, when the MUC16 expressing 3T3 cell lines (vector phrGFP, MUC16^c114^-GFP and MUC16^c344^-GFP) were implanted into the flanks of athymic nude mice, both the MUC16^c114^-GFP and MUC16^c344^-GFP formed larger tumors compared to the vector only controls at 4 weeks. There was not a statistical difference between the cell line expressing the MUC16^c114^-GFP and MUC16^c344^-GFP proteins (Fig 2E), suggesting the oncogenic effects of MUC16 expression are primarily linked to the most proximal parts of the molecule. This increase in tumor growth rate was seen throughout the experimental period of tumor growth and is consistent with the clinical linkage between high levels of MUC16 expression (as serum CA125) and poor survival [19].

### A2780 Human Ovarian Cancer Cells

**Fig 3:**
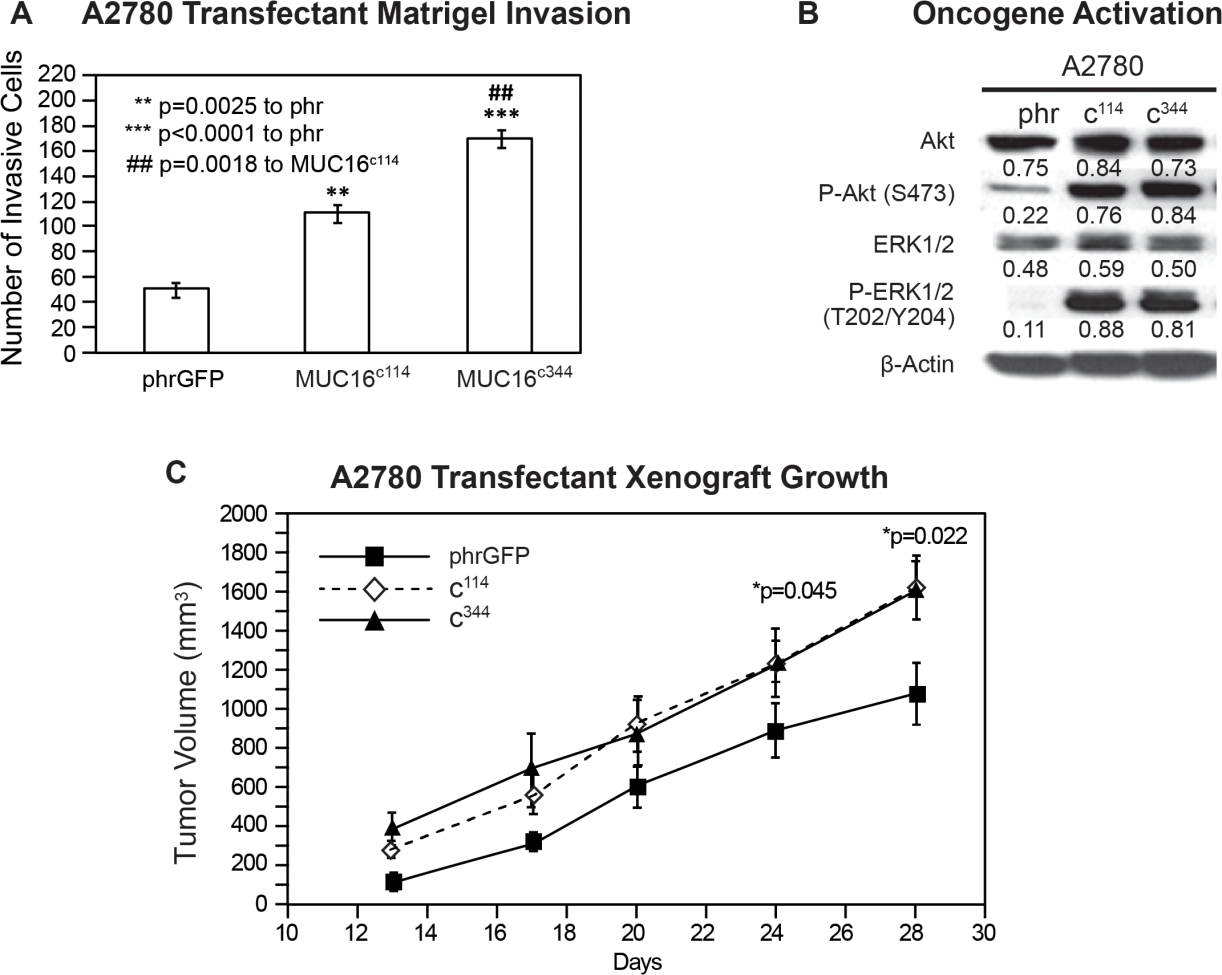
Effect of MUC16 in A2780. A) Matrigel invasion assay for A2780 cell lines transfected with either phrGFP control vector or with MUC16 carboxy-terminal expression vectors MUC16^c114^-GFP and MUC16^c344^-GFP. Each assay was performed two or more times in triplicate and counted by hand. Results were compared to the phrGFP control, and MUC16^c114^ and MUC16^c344^ transfected cell lines showed significant matrigel invasion compared to the phrGFP vector control. The MUC16^c344^ cell line is also significant (## p = 0.0018) compared to the MUC16^c114^ cell line. B) Effect of MUC16 expression on ERK/AKT signaling. A2780 cells were examined for activation of the ERK/AKT signaling pathways. Phosphorylation of ERK1/2 (pT202/Y204) and AKT (S473) was increased following expression of each of the MUC16 expression constructs. As with 3T3, activation of both pathways was seen in each of the MUC16 transfected cell lines. β-Actin normalized densitometry quantification values are shown below each Western blot. C) MUC16-positive tumor growth in athymic nude mice. Two million tumor cells were introduced into the flank of 15 nu/nu mice, and the mice were observed for tumor formation. Tumors were measured by calipers twice weekly. The differences in mean tumor volume were significantly greater for mice bearing any of the MUC16^c114^ or MUC16^c344^ tumors at day 28, as indicated in the figure.

While the expression of MUC16 protein in 3T3 cells was clearly linked to hallmarks of transformation, some fully transformed ovarian cancer cell lines lack MUC16 expression when cultured. In order to explore the contribution of MUC16 to the behavior of human ovarian cancer cells, we also transfected A2780 cells with the same MUC16 expression vectors, MUC16^c114^-GFP and MUC16^c344^-GFP. The MUC16 expression cells were selected by G418 and subjected to FACS for MUC16 and GFP expression. As with the 3T3 cells, the MUC16 expression did not alter growth on plastic with either 10% or 1% serum supplementation. Since these cancer cells grow well in soft agar, even in the absence of MUC16 expression, we went directly to the effect of the MUC16^c344^ on matrigel invasion. As shown in Fig 3A, MUC16^c114^ and MUC16^c344^ expression clearly promoted matrigel invasion in A2780 cells. As with the 3T3 cells, MUC16^c344^ A2780 cells were even more invasive than the MUC16^c114^ A2780 cell line, but even the MUC16^c114^ A2780 was much more invasive than the A2780 cells lacking MUC16. The effect of MUC16 on the activation of the ERK and AKT pathways was also similar to that seen in the 3T3 cells, increasing the basal levels of both pAKT(S473) and pERK (T202/Y204) (Fig 3B). Thus, even in the malignant ovarian cell lines, forced expression of MUC16 carboxy-terminal elements was strongly linked to increased matrigel invasion and oncogene activation. As a final confirmation, we examined the *in vivo* tumor growth of MUC16 transfected A2780 lines. These results are shown in thapi. 3C. The MUC16-positve cell lines with as little as 114 amino acids of the carboxy-terminal portion of MUC16 up to the full 344 amino acid expression grew more rapidly than the vector only controls. As with the 3T3 cell lines, the MUC16^c344^ and the MUC16^c114^ expression had a very similar effect on *in vivo* growth in the A2780 cells, but both transfected cell lines were more aggressive than the vector controls.

### Glycosylation studies

**Fig 4:**
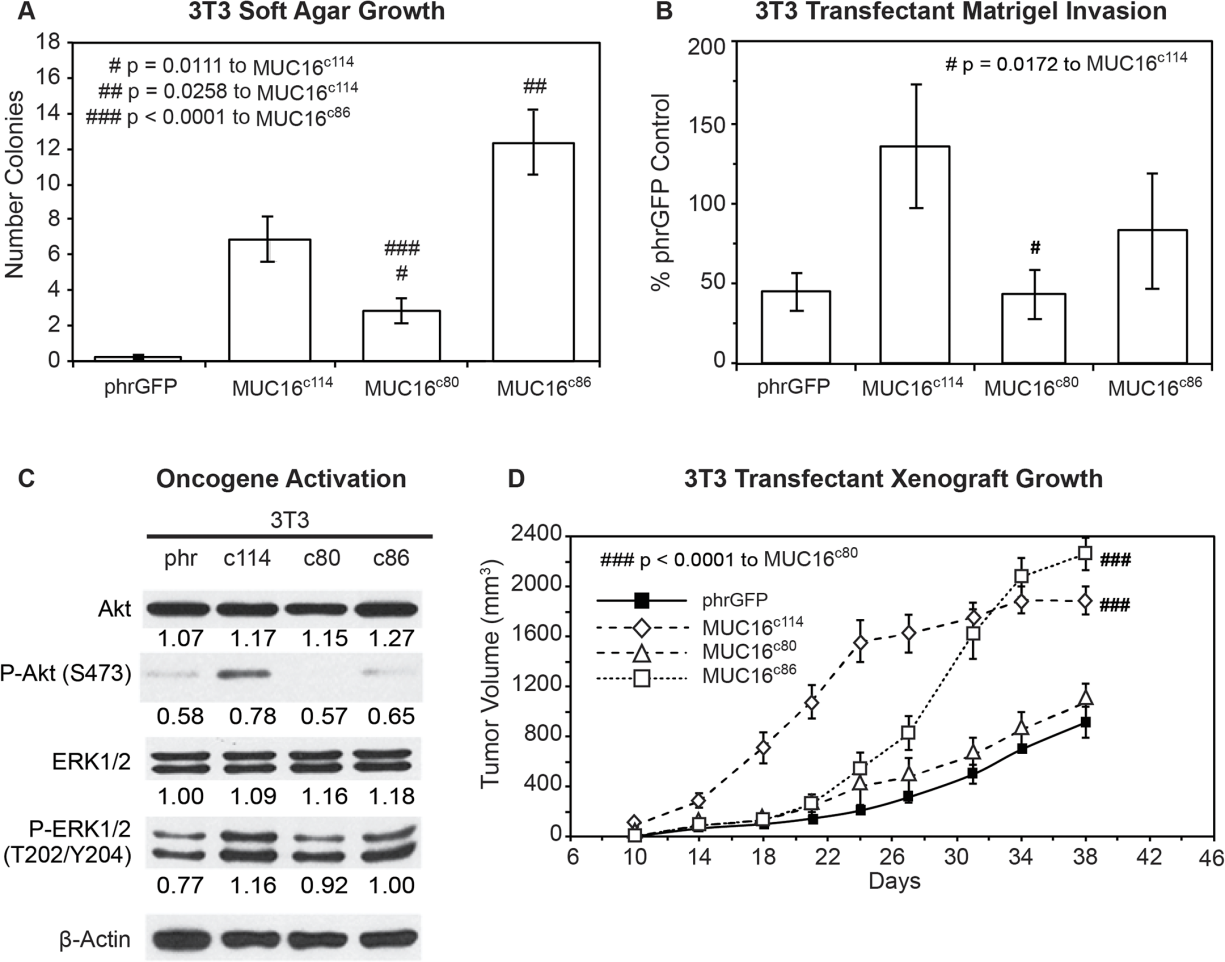
Effects of truncated MUC16^c114^ variants. A) Soft agar growth. 3T3 transfectants expressing either internal or external domain portions of MUC16^c114^ (S1 Fig) were layered on soft agar, as described in the Material and Methods section. Colonies were counted and plotted. The data shown represent one of three experiments. Soft agar growth rates for MUC16^c80^ and MUC16^c86^ were significant (# p = 0.0111 and ## p = 0.0258, respectively) compared to MUC16^c114^, whereas a higher level of significance (###p<0.0001) was seen with MUC16^c80^ transfectant compared to MUC16^c86^. B) Matrigel invasion assay for 3T3 cell lines transfected with either phrGFP control vector or with MUC16 carboxy-terminal constructs. Each assay was performed two or more times in triplicate and counted by hand. MUC16^c80^ transfectant was significant (# p = 0.0172) compared to the MUC16^c114^ cell line. C) Effect of MUC16 expression on ERK/AKT signaling. Transfected 3T3 cells were examined for activation of the ERK/AKT signaling pathways. Phosphorylation of ERK1/2 (pT202/Y204) and AKT (S473) was increased following MUC16^c114^ transfection; however, the signals were lower with either the MUC16^c80^ or MUC16^c86^ constructs. β-Actin normalized densitometry quantification values are shown below each Western blot. D) MUC16-positive tumor growth in athymic nude mice. Two million tumor cells were introduced into the flank of 20 nu/nu mice, and the mice were observed for tumor formation. Tumors were measured by calipers twice weekly. The differences in mean tumor volume were significantly greater for mice bearing MUC16 ectodomain positive tumors. 3T3 MUC16^c114^ and 3T3 MUC16^c86^ transfectants were significantly different compared to MUC16^c80^ (### p<0.0001) and vector only animals.

While the most proximal 114 amino acid fragment of MUC16 was sufficient to transform 3T3 cells and enhance the invasion potential of the A2780 human ovarian cancer cells, the minimal part of the 114 amino acid MUC16 protein fragment responsible for transformation was still uncertain. In order to explore this further, we devised two additional constructs: 1) a c80 construct that deleted a 34 amino acid sequence from the ectodomain of MUC16 (from position 1798 to 1831, as numbered in the original publication), including the 4H11 epitope [6], and 2) a c86 construct that retained the entire ectodomain of MUC16 but removed a 28 amino acid sequence from the cytoplasmic domain, including part of the putative EZRINbinding domain, the potential tyrosine phosphorylation sites, and SH2 domain (from position 1857 to 1884) (the sequences are detailed in Fig 1C and S1A Fig). These constructs were introduced into 3T3 cells and selected by FACS analysis for cell surface expression of the remaining MUC16 sequences and GFP. These two additional cell populations were then examined for the same MUC16-dependent changes we had previously studied. The MUC16^c86^ 3T3 cells with the intact “ectodomain” between the membrane and the putative cleavage site retained a much greater capacity for soft agar colony formation than the MUC16^c80^ 3T3 cells, which retained an intact cytoplasmic domain (Fig 4A) but lacked nearly all of the ectodomain. The soft agar colony formation of the MUC16^c80^ 3T3 cell line was significantly reduced compared to the MUC16^c114^ 3T3 cells while the soft agar colonies of MUC16^c86^ cells were increased compared to the MUC16^c114^ control value. This was also true of the capacity for matrigel invasion (Fig 4B). The MUC16^c80^ 3T3 cells, which lacked the ectodomain, had a rate of invasion that was statistically lower than that of the MUC16^c114^ 3T3 cells and similar to the vector only control lacking any MUC16 sequence. In contrast, the MUC16^c86^ 3T3 cells expressing the unchanged MUC16 ectodomain retained a more invasive phenotype, similar to the intact MUC16^c114^ and MUC16^c344^ cells. When the activation of AKT and ERK pathways was examined, the central role of the ectodomain on oncogene activation was consistent with the importance of the ectodomain in soft agar colony and matrigel invasion studies (Fig 4C). Those MUC16^c114^ elements that increased invasion were the same elements that increased AKT and ERK phosphorylation. Expression of the MUC16^c80^ fragment (without most the intact ectodomain) did not activate ERK and AKT and was similar to the phrGFP vector control in the Western blot. In contrast, the MUC16^c86^ expressing3T3 cells with the intact ectodomain were similar to the full MUC16^c114^ expressing 3T3 cells in the activation of ERK and AKT. Finally, the importance of the intact ectodomain was confirmed in the xenograft tumor models. Loss of the intact MUC16 ectodomain in the MUC16^c80^ 3T3 cells resulted in loss of MUC16^c114^ dependent 3T3 growth enhancement, while the MUC16^c86^ expressing 3T3 cells had a modest growth delay compared to the MUC16^c114^ 3T3 cells but had a statistically similar overall effect to the MUC16^c114^ expressing 3T3 cells by 30 days (Fig 4D). The control 3T3 cells and the MUC16^c80^ 3T3 cells had much slower growth and the rate of tumor growth for these two lines was indistinguishable in the mice.

**Fig 5:**
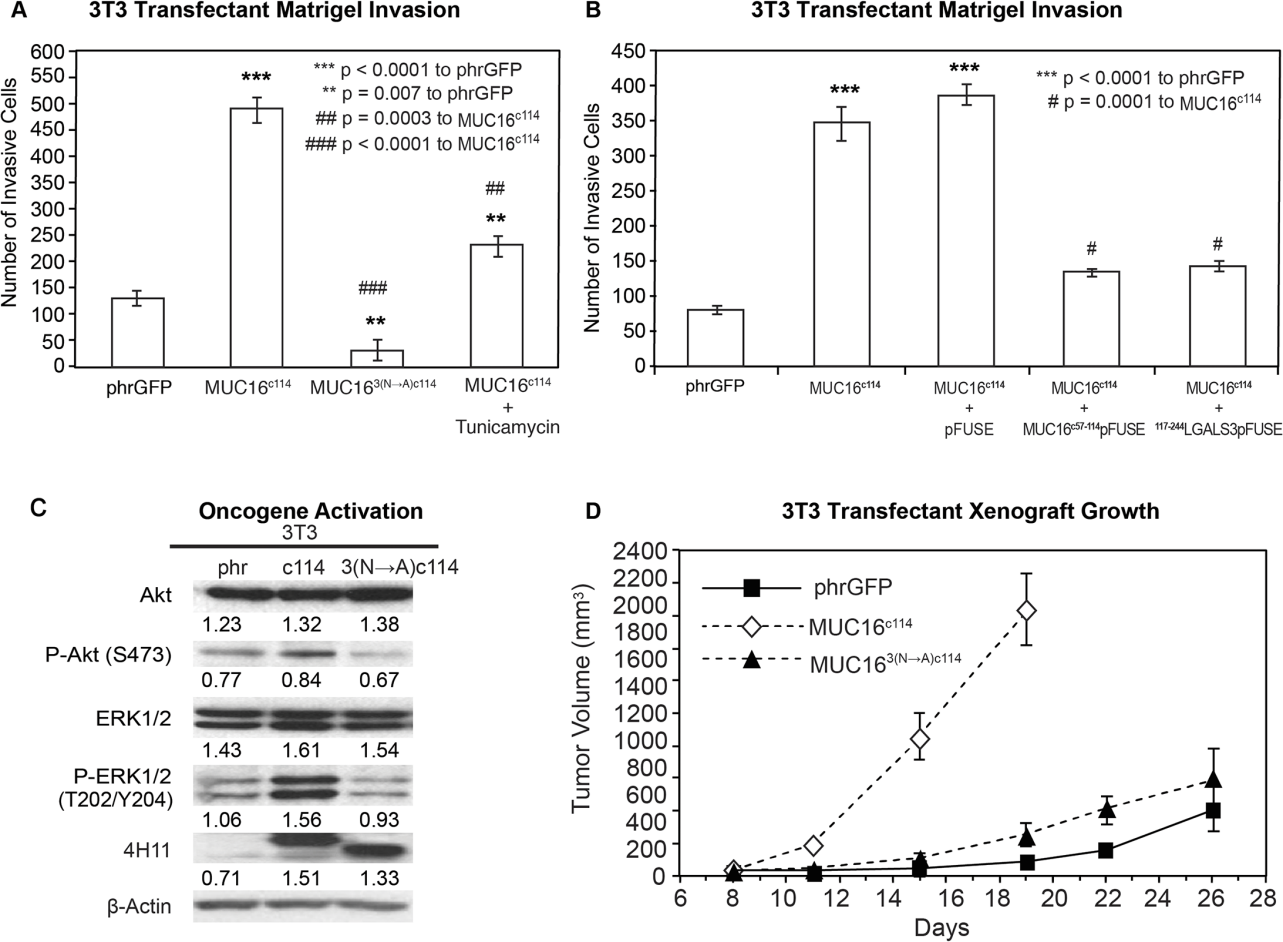
Effect of N-glycosylation on MUC16 transformation. A) Matrigel invasion assay for 3T3 transfected cell lines, phrGFP control vector or MUC16^c114^ or MUC16^3(N—A)c114^ and MUC16^c114^ treated with 0.1 μg/mL Tunicamycin. As seen earlier, MUC16^c114^ cell lines were significantly different (p<0.0001) than the phrGFP vector control. The MUC16^3(N—A)c114^ cell line was still significantly more invasive (** p = 0.007) compared to the phrGFP vector control. Treatment with the N-glycosylation inhibitor Tunicamycin significantly inhibited matrigel invasion compared to the untreated MUC16^c114^ (##p = 0.0003), and MUC16^3(N—A)c114^ is highly significant (### = p<0.0001) compared to MUC16^c114^, suggesting that N-glycosylation is critical for MUC16-induced matrigel invasion. B) Matrigel invasion assay for 3T3 transfected cell lines compared to the phrGFP control vector. 3T3 cells transfected with MUC16^c114^ were treated with media alone, with 5 μg/mL of control pFUSE hIgG1-Fc2 fusion protein, with 5 μg/mL of MUC16^c57-114^-pFUSE hIgG1-Fc2 fusion protein, or with 5 μg/mL of ^117-244^LGALS3-pFUSE hIgG1-Fc2 fusion protein, as detailed in S4 Fig As seen earlier, the MUC16^c114^ cell line was much more invasive than the phrGFP vector control 3T3 cells (p<0.0001), and this increase in invasion was unaffected by exposure to pFUSE vector only protein. In contrast, MUC16^c114^ cell line treated with MUC16^c57-114^-pFUSE hIgG1-Fc2 fusion protein or ^117-244^LGALS3-pFUSE hIgG1-Fc2 fusion protein demonstrated significant (# p = 0.0001) inhibition of matrigel invasion compared to MUC16^c114^ control cells. C) Effect of MUC16 expression on ERK/AKT signaling. Phosphorylation of ERK1/2 (pT202/Y204) and AKT (S473) was increased in the 3T3 transfected with MUC16^c114^; however, the effect was much diminished in 3T3 cells transfected with the MUC16^3(N—A)c114^ vector. Despite the three asparagine—> alanine mutations, Western blot with the anti-MUC16 antibody, 4H11 mAb, showed a higher signal than either the phrGFP vector control or the native MUC16^c114^ transfected cells, indicating that the high levels of MUC16^3(N—A)c114^ protein is expressed in the transfected 3T3 cells, and surface expression was confirmed in S2 Fig β-Actin normalized densitometry quantification values are shown below each Western blot in the figure. D) MUC16-positive tumor growth in athymic nude mice. Two million tumor cells were introduced into the flank of 20 nu/nu mice, and the mice were observed for tumor formation. Tumors were measured by calipers twice weekly. The differences in mean tumor volume were significantly greater for mice bearing MUC16^c114^ tumors (p<0.0001). As seen earlier, 3T3 MUC16^c114^ transfectant was highly significant at *** p<0.0001 compared to the phrGFP control vector. However, MUC16^3(N—A)c114^3T3 transfectants did not show any significance over phrGFP vector control 3T3 cells, indicating that the mutations of N-glycosylation dramatically decreased *in vivo* tumor growth and invasion.

Thus, the extracellular part of the MUC16^c114^ fragment was primarily responsible for the transformative effects of MUC16 in 3T3 cells. An additional 240 amino acids from the MUC16^c344^ had very modest effects, while loss of even 34 amino acids between the membrane and the cleavage site abrogated MUC16 oncogenic behaviors. In order to examine this further, we performed co-precipitation studies with the MUC16^c114^ expressing 3T3 cell, using our unique panel of MUC16-targeting antibodies [13]. No co-precipitating single bands were identified by silver staining, and specific Western blots for EGFR, integrin family members, and HER3 were negative (data not shown). However, further analysis of the MUC16^c114^ sequence suggested that the three potential N-glycosylation sites (N1777, N1800, and N1806 [S1A Fig]) in the ectodomain might play a role. We subsequently tested the role of N-glycoyslation in MUC16-driven transformation in several ways. Using site-specific point mutation, all of the asparagines were changed to alanines. This modified MUC16^c114^ construct was designated MUC16^3(N-A)c114^ and introduced into 3T3 cells, and MUC16^3(N-A)c114^ expressing cells were isolated by FACS and 4H11 ectodomain antibodies. As shown in Fig 5A, loss of N-glycosylation by these 3(N-A) mutations completely abrogated the MUC16^c114^ induced enhancement of matrigel invasion seen with the parent MUC16^c114^ expression vector in 3T3 cells. To confirm the role of N-glycosylation, the 3T3 cells were treated with the N-glycosylation inhibitor Tunicamycin (0.1 μg/mL), and a significant decrement in matrigel invasion was also noted. Either of two interventions decreased MUC16^c114^ induced matrigel invasion, and the complete loss of N-glycosyation by mutation reduced invasion below the amount of matrigel invasion seen in the 3T3 vector controls. Two other potential inhibitors of N-glycosylation effects were also examined to further explore the role of the MUC16 extracellular sequence. The MUC16 external sequence (from position 1777 to1834 as numbered in the original publication) [6] (Fig 1C and S1A Fig) was attached to a human Fc backbone pFUSE (MUC16^c57-114^ pFUSE) to provide a “dummy” receptor of the MUC16 ectodomain (S4A and S4B Fig). Dummy receptor strategies have been previously employed to inhibit interactions between proteins and their presumptive ligands. This construct was compared to both the MUC16^c114^ invasion and a pFUSE vector control lacking the MUC16 sequence. As shown in Fig 5B, the MUC16 ectodomain dummy receptor construct, MUC16^c57-114^ pFUSE, markedly diminished the overall effect of the MUC16^c114^ expression vector on matrigel invasion. In contrast, the pFUSE control had no effect on MUC16^c114^ dependent invasion. Based on the sensitivity of MUC16c114 transformation to alterations in N-glycosylation, we hypothesized that the effects were mediated by lectins. Galectin 3 (LGALS3) is over-expressed in ovarian cancer, and so a second protein inhibitor was constructed from the sugar-binding domain of LGALS3 (amino acids 117 to 244; S4C and S4D Fig) attached to the same pFUSE backbone (^117-244^LGALS3pFUSE) [25]. Like Tunicamycin, the Galectin 3 based protein inhibitor, ^117-244^LGALS3pFUSE, appeared to completely block MUC16^c114^ invasion, while the pFUSE vector alone had no effect (Fig 5B). As with other interventions, mutation of the N-glycosylation sites on MUC16^c114^ diminished pAKT and pERK expression in parallel with the loss of matrigel invasion when the N-glycosylation sites were removed, as shown in Fig 5C. However, the MUC16^3(N-A)c114^ construct had high levels of expression of the MUC16^3(N-A)c114^ protein, as demonstrated by 4H11 (MUC16 ectodomain specific) binding in FACS (S2 Fig). The impact of N-glycosylation mutation-loss was likewise confirmed in the reduction of growth in the transfected 3T3 cells in nu/nu mice, as shown in Fig 5D. Without the three N-gylycosylation sites, MUC16^3(N-A)c114^ did not alter 3T3 tumor growth over the vector control.

### Transgenic Mouse

**Fig 6:**
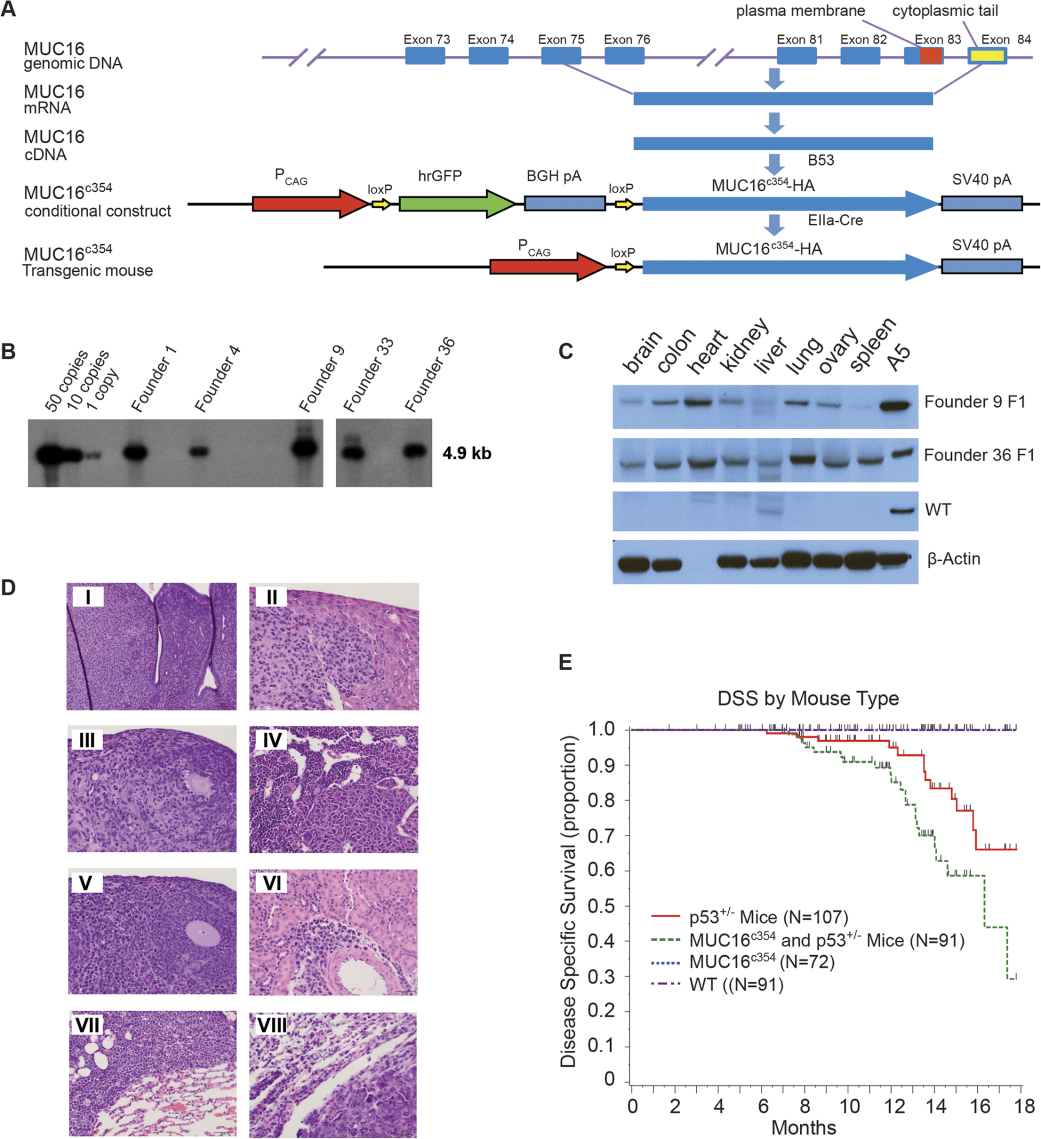
MUC16^c354^ transgenic mice. A) Strategy for MUC16^c354^ conditional construct. A CMV early enhancer plus the chicken β actin promoter (CAG) was used to drive the transcription of hrGFP between two loxPs and the downstream MUC16^c354^ sequence. B) Southern blot shows 12 candidates of MUC16^c354^ positive founders among 99 animals after the microinjection procedure. C) Western blot with anti-MUC16^c114^ 4H11 was used to identify founders 9 (~50 copies) and 36 (~10 copies) for MUC16^c354^ mouse colony development. A5 is a positive control from a stable transfected SKOV3 with MUC16^c354^. D) Histological analyses of tumors from double MUC16^c354^:p53^+/-^ transgenic mice. Multiple sarcomas and lymphomas were identified in the double MUC16^c354^:p53^+/-^ transgenic mice. Sections were stained with hematoxylin and eosin (H&E). Tumors included histocytic sarcoma in the uterus (I, Scale bar:100μm), liver (II, Scale bar:50μm), ovary (III, Scale bar:50μm) and bone marrow (IV, Scale bar:50μm); lymphoma in the ovary (V, Scale bar:50μm), kidney (VI, Scale bar:50μm), and lung (VII, Scale bar:50μm); and carcinoma in the lung (VIII, Scale bar:50μm). E) Transgenic mouse cancer-specific Kaplan-Meier survival curves: the MUC16^c354^ mice (black line) showed no spontaneous tumor development over the first 18 months, similar to the wild type (WT, red dashed line). However, when MUC16^c354^ mice were crossed with p53^+/-^ mice, the double transgenic MUC16^c354^:p53^+/-^ mice (green dashed line) showed a significantly worse overall survival due to spontaneous tumor development compared to either the p53^+/-^ mice (red line) (p<0.014) or the MUC16^c354^ mice. The number of tumors were p53^+/-^ mice 20/107; MUC16c354 and p53^+/-^ Mice 34/91; MUC16c354 1/72; and wild type 0/91.

Since the *in vitro* transformation data were very compelling for short carboxy-terminal elements of MUC16, the effect of expression of the carboxy-terminal elements in transgenic mice and the rate of spontaneous tumor formation was examined. Through the MSKCC Mouse Genetics Core Facility, we were able to create conditional transgenic MUC16^c354^ mice expressing the full c114 sequence and the most proximal CA125 bearing tandem repeat. We hypothesized that MUC16 would not be a strong oncogene. It is well recognized that the murine female reproductive system differs substantially from the human system and tissue-specific ovarian promoters have been weak and relatively difficult to use in transgenic systems. Consequently, we chose the CMV early enhancer plus chicken β actin promoter (CAG) to force substantial MUC16^c354^ expression in all murine tissues as our first model system. The strategy for these mice is illustrated diagrammatically in Fig 6A.

Conditional transgenic animals were selected by Southern blot, as shown in Fig 6B, and crossed with EIIa-Cre mice to produce MUC16^c354^ transgenic founders. As shown in Fig 6C, two founders were chosen and a colony of MUC16^c354^ transgenic mice was created. As expected, the two founders highly expressed MUC16^c354^ in many organs, for example, the brain, colon, heart, kidney, liver, lung, ovary, and spleen. These mice appeared to have no effect from the widespread ectopic expression of MUC16^c354^, with normal ratios of male:female progeny, normal rates of fertility, and apparently normal life span, exceeding 2 years. Necropsy of two apparently healthy animals (one male and one female) from the control population and the MUC16^c354^ transgenic mice at 3-month intervals up to 1 year was only remarkable for mild/moderate uterine endometrial hyperplasia in older female mice, but the incidence and severity was not significantly different than the wild type controls. Selected tissues are shown in S5 Fig Only one spontaneous soft tissue tumor (sarcoma) was observed in the colony of more than 100 animals observed for a minimum of 2 years.

Based on this result, we hypothesized that a “second hit” would potentially be required. It is noteworthy that murine models of BRCA1 mutation also required a second hit, and loss of p53 significantly increased the frequency of tumors [26]. Human high-grade serous ovarian cancer is almost uniformly characterized by loss of p53 function. We crossed our MUC16^c354^ mice with p53^+/-^ mice from The Jackson Laboratory. There was limited early effect. However, after approximately 6 months, MUC16^c354^:p53^+/-^ mice began to develop spontaneous sarcoma tumors of the bone, soft tissue sarcomas, and lymphomas at a rate higher than that of normal control animals. The histology of selected tumors are shown in the panel insets of Fig 6D. The Kaplan-Meier survival for these mice is shown in Fig 6E. Nearly all of the observed deaths were apparently due to spontaneous tumor development, and no other histologic lesions were observed to suggest other morbid effects. The MUC16^c354^:p53^+/-^ mice showed a significantly worse overall survival due to spontaneous tumor development (p <0.014). The total number of tumors seen in each group were p53^+/-^ mice 20/107; MUC16^c354^ and p53^+/-^ mice 34/91; MUC16^c354^ 1/72; and wild type 0/91. When eight tumors were collected and examined for p53 genomic sequencing, all of the spontaneous tumors had loss of the normal allele of p53, indicating that MUC16-dependent tumor development also requires loss of normal p53 function. There were no uterine or ovarian tumors seen in either the MUC16^c354^ mice or the MUC16^c354^ and p53^+/-^ double transgenics.

### Ovarian TCGA

**Fig 7:**
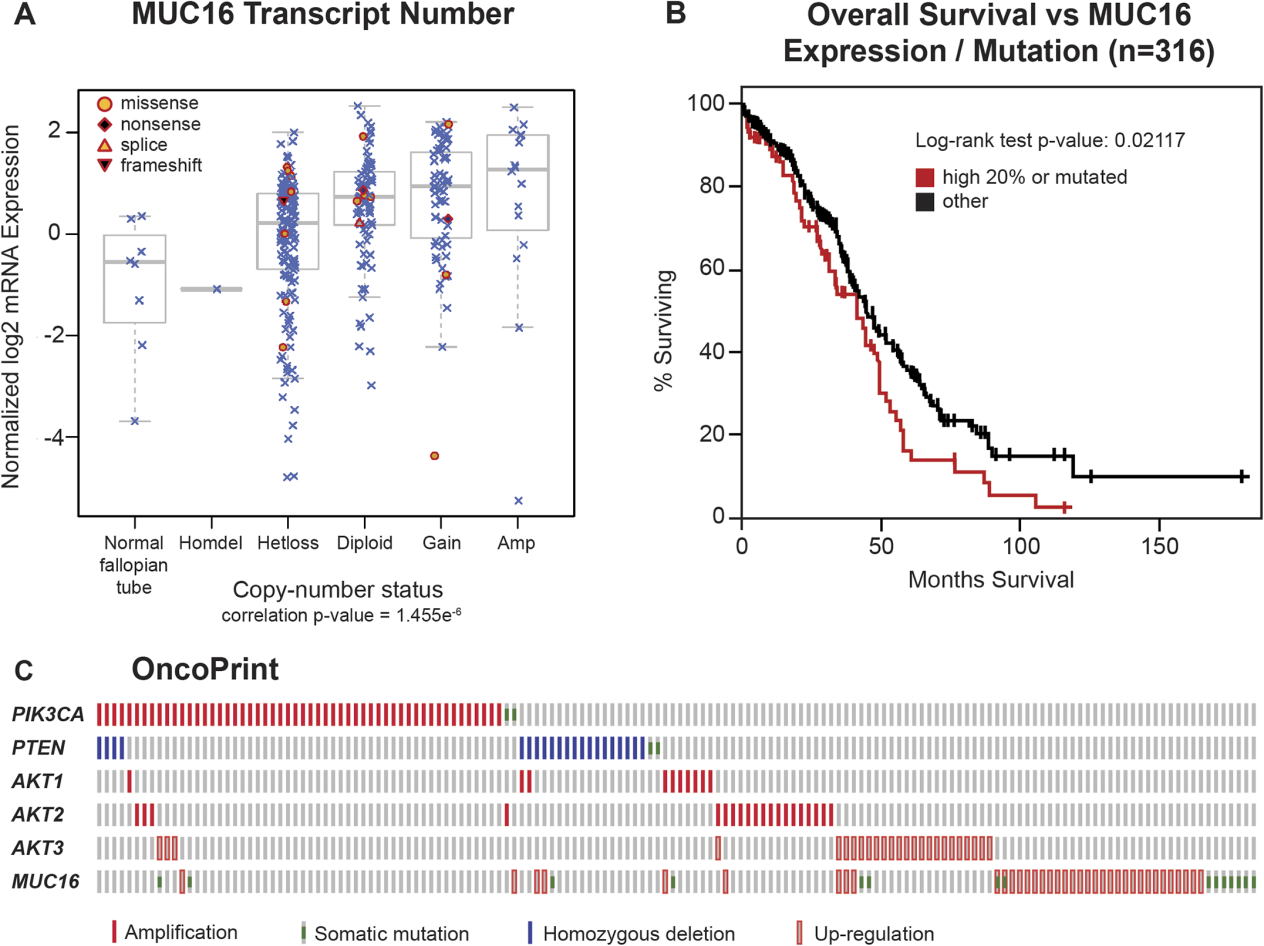
Impact of MUC16 in human ovarian cancer. A) MUC16 Transcript Number. The Cancer Genome Atlas ovarian cancer project is a well-studied collection that contains 316 serous ovarian cancers with complete data, including clinical outcome data. Since expression of MUC16 protein is an important driver of cancer behavior in ovarian cancer, we examined the impact of MUC16 copy number on MUC16 mRNA expression. The MUC16 transcript expression was related to the number of MUC16 gene copies, although there was a broad variation in MUC16 transcript expression in all of the groups examined (except, of course, the rare homozygous deletion of MUC16). In most cases, the MUC16 mRNA expression was clustered at higher transcript numbers than the normal fallopian tube samples included as controls. B) The quintile of patients with the highest MUC16 expression, combined with the 18 patients with identified MUC16 mutations, have a significantly (p = 0.02117) worse survival compared to the patients with lower MUC16 expression in a Kaplan-Meier analysis. C) An OncoPrint showing the relationship of MUC16 genetic alterations with PI3K mutational events in ovarian cancer.

The impact of MUC16 on transformation and tumor aggressiveness in the experimental models led us to re-examine the link between genetic alterations in MUC16 and the outcomes in ovarian cancer. The TCGA ovarian cancer project is a well-studied collection containing 316 serous ovarian cancers with complete data, including clinical outcome data. Since expression of MUC16 protein is an important driver of cancer behavior, we examined the impact of MUC16 copy number on MUC16 mRNA expression. The MUC16 transcript expression was generally related to the MUC16 gene copy number, although there was a broad variation in MUC16 transcript expression in all of the groups examined (except, of course, the rare homozygous deletion of MUC16). In most cases, the MUC16 mRNA expression was clustered at higher transcript numbers than the normal fallopian tube samples included as controls. Gene copy number is one of several variables that will potentially alter the expression of MUC16 protein, but it is clear that MUC16 mRNA expression is often increased in serous ovarian cancer (Fig 7A). We also examined the combined impact of MUC16 over-expression or mutation on clinical outcomes in the TCGA data set. When the TCGA data set is divided into MUC16 expression quintiles, the 20% of patients with the highest MUC16 expression had a significantly worse survival than the patients with lower MUC16 expression (p = 0.02969). This relationship was further strengthened when the 18 patients with MUC16 mutations were included in the high MUC16 expression group (p = 0.02117), as shown in Fig 7B. Taken together, this analysis demonstrates that MUC16 expression has an adverse impact on the survival of patients with ovarian cancer and confirms the negative biologic effects of MUC16 expression identified in our preclinical models. It is also consistent with the findings by Zorn et al who found a negative impact of serum CA125 levels [19].

Ovarian cancers often demonstrate activation of the PI3K pathway. These activations occur primarily through amplification and over-expression rather than point mutation events, as ovarian cancer is generally characterized by alterations in copy number. Based on the activation of the PI3K/AKT pathway in our cell line models, we also examined the relationship between MUC16 and other activating genetic alterations in the PI3K pathway. As shown in the Fig 7C OncoPrint, over-expression and mutation events associated with MUC16 are generally complementary with other pathway events like PTEN loss, and amplification of AKT1, AKT2, or PI3KCA. The mechanism of this MUC16-driven AKT activation remains unknown. We also examined the role of ERK activation, but no link between MUC16 expression and ERK pathway mutations was identified.

## Discussion

MUC16, encoding the CA125 antigen, circulates in the plasma of many patients with high-grade serous ovarian cancer [1]. MUC16 is unique among the tethered mucins for its limited expression outside mullerian tissues [3, 13]. While increasingly viewed as an adverse prognostic factor independent of tumor bulk, the biologic mechanism for its negative impact has not been well understood [19]. The NH_2_-portion of the molecule contains multiple tandem repeats that encode the CA125 antigen and appear to serve as important adhesion partners to mesothelin and some galectins (Fig 1A) [6, 16, 27–29]. While these adhesion functions have been suggested to be critical in MUC16-related biology and adverse outcome, these studies do not explain all of the observed changes in ovarian cancer cell behavior. The cloning of the MUC16 glycoprotein has provided basic structural information about the MUC16 gene product [4, 6]. These observations are among the first data to indicate that MUC16 may mediate signaling from the environment into the cancer cell. In particular, we have identified the glycosylated MUC16 ectodomain as critical to MUC16 alterations in cancer cell behavior.

In these studies, we have demonstrated that the 114 amino acids from the most proximal carboxy-terminal portion of MUC16 are sufficient to transform NIH/3T3 (3T3) cells, supporting both increased soft agar growth and increased matrigel invasiveness. While others have identified the most proximal portion of MUC16 as the critical elements in MUC16-induced behaviors, we link these behaviors to the N-glycosylation sites in the retained MUC16 ectodomain. These changes are associated with an altered gene-expression profile and increased expression of critical invasion genes such as MMP2, MMP9, CXCL12, and CDH11. While longer elements may induce virulent behavior, even the residual 114 amino acids proximal to the putative cleavage site are sufficient in 3T3 cells to induce the same changes in invasion gene expression. These findings are most consistent with an “outside in” signal transduction by the most proximal portions of the protein, including a residual extracellular domain along with the transmembrane domain and cytoplasmic tail. In our hands, loss of the intracellular cytoplasmic domain has less impact than loss of the glycosylated ectodomain, in contrast to the results of Theriault and Giannakouros [18, 30]. These differences may reflect the specific mutations chosen and the methodology to reduce expression [18, 30]. For example, loss of N-glycosylation trafficking signals or impaired EZRIN domain function might alter the observations in different experimental systems. The MUC16-dependent “inside-out” signal appears to activate a transcription of a gene program that facilitates the implantation and growth of MUC16-expressing cells in soft agar and nude mice. When the transfected cells are examined for activation of common oncogenic pathways, both AKT and ERK pathways appear to be activated by constitutive expression of MUC16. The mechanism by which MUC16 increases AKT/ERK phosphorylation is unclear and will require further studies. It is possible that orthotopic, intraperitoneal ovarian cancer models may give additional insight into this problem through exploration of cancer-stromal interaction. The absence of co-precipitating receptors suggests that other, more novel mechanisms may also be involved. Although MUC16 sequences are very different, other tethered mucins, including both MUC1 and MUC4, have been shown to act as signal-generating oncogenes in 3T3 cells and rat fibroblasts [8, 9]. It is likely that the role of mucins on the cancer cell surface play important roles through mechanisms that are still being defined.

Based on the findings in 3T3 cells, the results of the MUC16 transgenic mouse experiment is highly supportive. By itself, the same MUC16 proximal 354 sequence could be readily expressed in nearly all murine tissues with no adverse effect in the transgenic mouse. The rate of spontaneous tumor formation was very low in those mice, and reproductive function seemed unaffected. However, like other murine ovarian cancer models, loss of p53 function appears to play a strong permissive role in MUC16-dependent tumor formation [31]. These results certainly are consistent with uniform p53 inactivation, which characterizes ovarian cancer in the TCGA data set.

These findings are the first to describe MUC16-linked changes in cellular behavior and gene transcription. The *in vitro* and *in vivo* models are consistent with the adverse effects of MUC16 expression levels in serous ovarian cancer and promote the understanding of MUC16 as a pathogenic contributor to the behaviors of ovarian cancer. The adverse impact of increasing CA125 expression is consistent with increased *in vivo* tumor growth and lethality of MUC16-positive 3T3 transfectants. Additional work of the precise mechanisms initiating MUC16 signaling, the effects of MUC16 binding to stromal elements, and the identification of the key molecular partners of cell surface MUC16 are likely to further enhance our understanding of ovarian cancer biology.

## Material and Methods

### Synthesis of MUC16 Carboxy-Terminal (MUC16^c114^) and MUC16-CA125 Domain (MUC16^c344^) DNA Constructs and Glycosylated Fusion Protein

EcoRV and NotI multiple cloning sites of the phrGFP II-C vector (phrGFP) (Stratagene, LaJolla, CA) were used to incorporate MUC16^c114^, MUC16^c80^, MUC16^c86^, and MUC16^c344^ DNA so that MUC16-GFP fusion constructs were obtained with the GFP protein present on the carboxy-terminal portion of the fusion protein. Polymerase chain reaction (PCR) products for the MUC16 fragments were created using pBK-CMV-MUC16-B53 DNA as a template (kindly provided by Dr. Beatrice Yin and Dr. Ken Lloyd, MSKCC, New York, NY), and PCR products were purified in a 1% agarose gel, sequenced, and inserted into the phrGFP II-C vector (phrGFP). The pFUSE-hIgG1-Fc2 vector was purchased from InvivoGen (San Diego, CA). PCR primers were designed for the ectodomain MUC16^c57-114^ (from position 1777 to1834, as numbered in the original publication) [6] (S4A Fig), or the sugar-binding domain of ^117-244^LGALS3 DNA sequences (S4C Fig) were synthesized (Sigma-Genosys, The Woodlands, TX) with the restriction enzyme site EcoRV as the forward primer and the restriction enzyme site NcoI as the reverse primer. PCR products for the MUC16^c57-114^ fragment were created using pBK-CMV-MUC16-B53 DNA as a template and LGALS3 cDNA clone (MGC:2058 IMAGE:3050135 GenBank: AAH01120.1; DBSource accession BC001120.2), which was obtained from the American Type Culture Collection (ATCC; Manassas, VA) [25] and was used as a DNA template to synthesize the sugar-binding domain of the LGALS3 PCR product. PCR products were purified in a 1% agarose gel, sequenced, and inserted into the pFUSE-hIgG1-Fc2 vector.

### Cell Cultures

The NIH/3T3 (3T3) cells were obtained through the ATCC, and the A2780 cells [32] were obtained as a gift from Dr. Thomas Hamilton (Fox Chase Cancer Center, Philadelphia, PA). Both cell lines were maintained according to published conditions. For the creation of MUC16-positive transfected cell lines, stable cell lines were created by transfection of MUC16 expression vectors and selected using geneticin (G418, Invitrogen, Grand Island, NY) in their respective culture media and isolated by expression of GFP. The characteristics of the MUC16 transfectants are described elsewhere and summarized in the supporting information (S1 Data) [13]. The MUC16^c114^ transfectants have cell surface expression of MUC16 protein from the putative cleavage site to the carboxy-terminal portion (amino acids 1777 to 1890) [5]. Cell lines with longer MUC16 fragments were prepared in a similar manner, including lines with expression of MUC16^c344^—GFP vector that have cell surface expression of MUC16 protein as a 344 amino acid fragment extending to the carboxy-terminal portion of MUC16 (amino acids 1547 to 1890).

### Transfection

All of the constructs were introduced into NIH/3T3 (3T3) and A2780 cells using DOTAP (Roche Diagnostics, Indianapolis Corporation, IN) following the manufacturer’s protocol. Stable transfectants were selected with 400 μg/mL of G418 for 3T3 and A2780 cells in their respective culture media. They were cell sorted twice for GFP expression and MUC16 expression at the MSKCC Flow Cytometry Core Facility (FCCF), and selected cells were grown as lines of up to 15 passages. Routine monitoring of FACS analysis was done to confirm the GFP positivity of these lines. Protein extracts of these lines were analyzed by Western blot using anti-hrGFP (Stratagene, La Jolla, CA) and anti-MUC16-carboxy-terminal monoclonal antibodies [5].

### Growth Curves

One thousand stable transfected cells/well were seeded in 200 μL of culture media/well in multiple 96 well flat bottomed plates and incubated at 37°C and 5% CO_2_ for 5 days. Every day, triplicate cultured plates were developed with 25 μL/well of Alamar Blue (ABD Serotec Co. UK) and incubated at 37°C and 5% CO_2_ for 4 hours. Plates were read on PerSeptive Biosystems CytoFluor Multiwell Fluorescent Plate Reader Model # 4000 with excitation at 530 nM and emission at 620 nM. Growth curves over 4 days were recorded, and the mean values from triplicate plates were plotted accordingly.

### Soft Agar Assay

Stable transfected cells were placed in an agarose suspension and plated over a thin agarose layer and analyzed for their ability to form anchorage independent colonies. One to 5 million cells in 10 mL of media-agarose suspension were plated per dish and incubated at 37°C and 5% CO_2_. The plates were monitored for colony formation. Additional culture media were overlaid every 4–5 days. After 11–14 days of culture, colonies were enumerated, and pictures of the colonies were taken.

### Transfection of Eukaryotic Expression Vectors

The MUC16^c57-114^-pFUSE-hIgG1-Fc2 and ^117-244^LGALS3-pFUSE-hIgG1-Fc2 constructs were separately transfected into human embryonic kidney (HEK) FreeStyle 293F cells (Invitrogen, CA) that express and secrete fusion proteins into serum-free media, as per the manufacturer’s protocol. Secreted fusion proteins were purified and characterized by Western blot analysis using anti-human IgG1-Fc-HRP (γ1 chain specific) (Southern Biotech Inc., Birmingham, AL) or 4H11-HRP or polyclonal anti-human LGALS3 antibody (Abgent, San Diego, CA) (S4B and S4D Fig).

### Invasion

Basement membrane invasion was determined in matrigel invasion chambers (BD Biosciences, Bedford, MA). Matrigel migration was measured at 48 hours in triplicate wells and compared with phrGFP vector controls or MUC^c114^ and MUC16^c86^ transfectants. 0.1 μg/mL of Tunicamycin (Sigma-Aldrich, St. Louis MO cat # T7765) or 5 μg/mL of MUC16^c57-114^pFUSE-hIgG1-Fc2 or 5 μg/mL of ^117-244^LGALS3 pFUSE-hIgG1-Fc2 fusion protein treated stable cell line matrigel migration after 48 hours was measured and compared with phrGFP Control and MUC16^c114^.

### Real-Time Polymerase Chain Reaction

RNA isolation was prepared by following the RiboPure Kit (Ambion, Austin, TX) protocol. RT PCR for a panel of metastasis and extracellular matrix protein genes was performed utilizing the RT^2^ Profiler PCR Array system (Super Array, Frederick, MD), as previously described [33].

### Tumor Growth in Athymic Nude Mice

Transfected cell lines and appropriate control cell lines were introduced into the flank or peritoneal cavity of athymic nude mice, and routine animal care was provided by the MSKCC Antitumor Assessment Core Facility. For tumor growth assessment experiments, 2 million cells from each tumor line were implanted into each of 5–15 athymic nude mice. Tumor measurements were taken twice a week, and tumor growth was recorded to a maximum size of 1500 mm^3^ or tumor ulceration, as per MSKCC Research Animal Resource Center guidelines

### Western Blot Analysis

Stable cell lines were cultured in 10 cm dishes in their respective culture media and incubated at 37°C and 5% CO_2_ for 3 days. They were then washed twice with ice cold PBS and scraped with 1–2 mL of ice cold PBS and centrifuged. The pelleted cells were lysed with 0.2 mL of modified Ripa lysis buffer (20 mM Tris-HCL, pH 7.4; 150 mM NaCl; 1% NP-40; 1 mM Na_3_VO_4_; 1 mM PMSF; 1 mM DTT; with protease and phosphatase inhibitors cocktails [cat # 11836170001 from Roche Diagnostics, IN]) for 30 minutes on ice and centrifuged at 4°C for 10 minutes. Protein concentration of the supernatant was measured using Bio-Rad Protein Assay (BioRaD Laboratories, Hercules, CA). Equal amounts of protein were separated by SDS-Poly Acrylamide Gel Electrophoresis (SDS-PAGE) and transferred to PVDF membrane using BioRad transfer apparatus at 4°C. The membranes were blocked with 3% bovine serum albumin (BSA) or 5% non-fat milk in PBS with 0.1% Tween-20 (PBST) at 4°C overnight. Membranes were developed with a variety of primary antibodies (Cell Signaling, MA: Akt cat #9272; Phospho-Akt (Ser473)(193H12) cat # 4058; p44/43 MAPK (Erk1/2) cat # 9102; Phospho- p44/43 MAPK (Erk1/2)(Thr202/Tyr204) cat #9101); (Sigma-Aldrich, Inc., St. Louis, MO: β actin cat # A5441); (Southern BioTech, Birmingham, AL: Anti-human-Fc-IgG1-HRP cat # 9054–05) and Abgent, San Diego, CA: Polyclonal LGALS3 antibody cat # AP11938b) at 4°C overnight. The membranes were washed three times with PBST, and developed with HRP-conjugated anti-mouse or anti-rabbit antibody (GE Healthcare, UK) (1:5000 dilution) for 1 hour at room temperature. Membranes were then washed three times with PBST and developed with a Western Lightning Chemiluminescence reagent (ECL, Perkin Elmer) for 1–5 minutes at room temperature, and the signals were developed on HyBlot CL film (Denville Scientific Inc. Metuchen, NJ).

### TCGA expression analysis of MUC16

Comprehensive genomic data were available for 316 serous ovarian cancer samples as part of the TCGA project (tcga.cancer.gov). Gene-level DNA copy-number calls were derived from CBS-segmented Agilent 1M microarray data using GISTIC. *MUC16* mRNA expression was measured using three different platforms (Agilent 244K Whole Genome Expression Array, Affymetrix

HT-HG-U133A, and Affymetrix Exon 1.0 arrays), and gene expression values were derived as previously described. Somatic mutations in *MUC16* were identified whole exome capture followed by next-generation sequencing (SOLiD or Illumina). All TCGA data were downloaded from the cBio Cancer Genomics Portal (www.cbioportal.org). mRNA expression values were then correlated with the corresponding DNA copy-number categories (homozygous deletion, hemizygous deletion, diploid, gain, high-level amplification), and somatic mutations were overlaid across all samples and plotted as a boxplot using the statistical framework R (www.R-project.org), as previously described [34]. Clinical data were obtained from the TCGA data portal (tcga-data.nci.nih.gov/tcga/).

### MUC16^c354^ transgenic mice

The conditional carboxy-terminal 354 amino acids (MUC16^c354^) transgenic construct was made using vector phrGFP II-C (Stratagene, La Jolla, CA), and CMV promoter was replaced with CAG promoter from vector pCAG-CreERT2 (Addgene, Cambridge, MA). MUC16^c354^ fragment was amplified by PCR from the construct B53 that was made by Yin BW, et al [5, 6]. The MUC16^c354^ conditional construct contains the following units: pCAG, 5’ loxP, hrGFP, BGHpA, 3’ loxP, MUC16^c354^, HA, and SV40pA.

Using the above MUC16^c354^ conditional transgenic construct, the MSKCC Mouse Genetics Core Facility performed the microinjection procedure on B6CBAF1/J mice. Twelve MUC16^c354^ conditional transgenic mice were identified from 99 mice by Southern blot. All 12 pro-founders were mated with B6.FVB-Tg(EIIa-cre)C5379Lmgd/J mice (The Jackson Laboratory, Bar Harbor, MI) to remove hrGFP, which was located between two loxPs. MUC16^c354^ PCR positive female mice for each pro-founder were dissected. The organs (brain, colon, heart, kidney, liver, lung, ovary, and spleen) from these dissected mice were minced and homogenized. The protein samples were analyzed by Western blot to identify the founders which highly express MUC16^c354^. The resulting transgenic mice were maintained on a mixed background.

We crossed two founders of transgenic MUC16^c354^ mice with p53 heterozygous mice (B6.129S2-Trp53^tm1Tyj^/J) (The Jackson Laboratory, Bar Harbor, MI) to create double transgenic MUC16^c354^:p53^+/-^. The resulting transgenic mice were maintained on a mixed background. All mice were genotyped by PCR using extracted toe or tail DNA. All experimental animals were maintained in accordance with the guidelines approved by the MSKCC Institutional Animal Care and Use Committee and Research Animal Resource Center and the NIH Guide for the Care and Use of Laboratory Animals. Please see the completed S1 ARRIVE Checklist.

### Histological analysis

Mice at 3–12 months of age were sacrificed and necropsied. Following macroscopic examination, dissected tissue samples were fixed for 24 hours in 10% neutral buffered formalin, then processed in alcohol and xylene, embedded in paraffin, sectioned at 5 μm thickness, and stained with hematoxylin and eosin (H&E). Tissues were examined by a veterinary pathologist (SM), and neoplastic and non-neoplastic lesions were diagnosed according to published guidelines on rodent pathology nomenclature.

### Statistical Analysis

Student’s two-sided paired *t* test was used to compare groups for studies of *in vitro* growth, invasion, and soft agar growth potential. The chi square test was used to analyze RT-PCR data for significance, according to provided software (SuperArray). The comparisons of the tumor volumes were made using area under the curve assessments for total tumor volume over time in each animal. The assessment of tumor volume was made based on the last day that all animals were alive in both groups. A non-parametric test for ranks (Wilcoxon two sample test) was used to test for a difference in distributions among the groups. In the animal survival studies, a time to event analysis was performed, with the event defined as time to tumor volume exceeding 1500 mm^3^ or ulceration. Animals with tumor volume less than 1500 mm^3^ were followed for up to 60 days and then censored whenever the last mouse from comparison groups were sacrificed. The Kaplan-Meier method was used to estimate survival distribution [35].

## Supporting Information

S1 ARRIVE Checklist This checklist describes our animal-based research in accordance with the guidelines approved by the MSKCC Institutional Animal Care and Use Committee and Research Animal Resource Center and the NIH Guide for the Care and Use of Laboratory Animals. (PDF)

S1 Data These data contain supporting Materials and Methods information, including a summarization of the characteristics of the MUC16 transfectants. (DOC)

S1 Fig MUC16^c114^ (1777 to 1890, Green Bar, S1A Fig) and MUC16^c344^ (1547 to 1890 Blue Bar and Green Bar, S1B Fig) amino acid sequences (Yin & Lloyd, 2001) [6] used in this study. The N-glycosylation sites are highlighted in dark green; the O-glycosylation site is highlighted in gray; and the transmembrane domain (1835 to 1859) is underlined. The 28 amino acid internal domain deletion (1857–1884) and the 34 amino acid ectodomain deletion (1798–1831) are noted in red letters.
(TIF)

S2 Fig FACS analysis showing geometric mean 4H11-PE fluorescence in S2Ai and S2Aii Fig) 3T3 transfectants and S2B Fig) A2780 transfectants. In each case, substantial MUC16 is present on the cell surface.
(TIF)

S3 Fig *In vitro* growth curves for MUC16 transfectants: A) 3T3; B) A2780; and C) 3T3 deletion mutant transfectants. Panels A and B include a GFP vector control, a MUC16^c114^-GFP minimal carboxy element, and a more extended expression vector. In each case, the growth is supported by 10% heat inactivated calf serum. The growth of 3T3 cells was reduced for all cell lines in media with 1% heat inactivated calf serum. The MUC16^c344^-GFP was introduced into 3T3 and A2780 cell lines. Panel C describes the *in vitro* growth of the deletion mutants. No statistical differences are seen among any of the curves.
(TIF)

S4 Fig S4A Fig) Ectodomain MUC16^c57-114^ (1777–1834 of MUC16) amino acid sequence inserted into the pFUSE-hIgG1-Fc2 vector to construct the MUC16^c57-114^pFUSE-hIgG1-Fc2 as a sham receptor. S4B Fig shows 293 cell expression of MUC16^c57-114^pFUSE-hIgG1-Fc2 fusion protein, Western blot. S4C Fig shows the ^117-244^LGALS3 amino acid sequence inserted into pFUSE-hIgG1-Fc2, resulting in the ^117-244^LGALS3pFUSE-hIgG1-Fc2 vector. S4D Fig shows 293 cell expression of ^117-244^LGALS3pFUSE-hIgG1-Fc2 fusion protein, Western blot.
(TIF)

S5 Fig Representative tissue from 12-month-old male and female MUC16^c354^ transgenic mice. Tissue sections were stained with hematoxylin and eosin (scale bar: 50 μm). Uterine endometrial hyperplasia was observed with similar incidence and severity in both genotypes (here only shown in the transgenic animal). The ovary, lung, colon and liver of transgenic animals (Tg) were similar to the parental line (wild type, WT).
(TIF)

## References

[1] Bast RC Jr., Badgwell D, Lu Z, Marquez R, Rosen D, Liu J, et al (2005) New tumor markers: CA125 and beyond. Int J Gynecol Cancer 15 Suppl 3: 274–281. 16343244 10.1111/j.1525-1438.2005.00441.x

[2] Bast RC Jr., Klug TL, St John E, Jenison E, Niloff JM, Lazarus H, et al (1983) A radioimmunoassay using a monoclonal antibody to monitor the course of epithelial ovarian cancer. N Engl J Med 309: 883–887. 6310399 10.1056/NEJM198310133091503

[3] Kabawat SE, Bast RC Jr., Welch WR, Knapp RC, Bhan AK (1983) Expression of major histocompatibility antigens and nature of inflammatory cellular infiltrate in ovarian neoplasms. Int J Cancer 32: 547–554. 6358053 10.1002/ijc.2910320505

[4] O'Brien TJ, Beard JB, Underwood LJ, Dennis RA, Santin AD, York L (2001) The CA 125 gene: an extracellular superstructure dominated by repeat sequences. Tumour Biol 22: 348–366. 11786729 10.1159/000050638

[5] Yin BW, Dnistrian A, Lloyd KO (2002) Ovarian cancer antigen CA125 is encoded by the MUC16 mucin gene. Int J Cancer 98: 737–740. 11920644 10.1002/ijc.10250

[6] Yin BW, Lloyd KO (2001) Molecular cloning of the CA125 ovarian cancer antigen: identification as a new mucin, MUC16. J Biol Chem 276: 27371–27375. 11369781 10.1074/jbc.M103554200

[7] Hollingsworth MA, Swanson BJ (2004) Mucins in cancer: protection and control of the cell surface. Nat Rev Cancer 4: 45–60. 14681689 10.1038/nrc1251

[8] Bafna S, Singh AP, Moniaux N, Eudy JD, Meza JL, Batra SK (2008) MUC4, a multifunctional transmembrane glycoprotein, induces oncogenic transformation of NIH3T3 mouse fibroblast cells. Cancer Res 68: 9231–9238. 10.1158/0008-5472.CAN-08-3135 19010895 PMC2610629

[9] Li Y, Liu D, Chen D, Kharbanda S, Kufe D (2003) Human DF3/MUC1 carcinoma-associated protein functions as an oncogene. Oncogene 22: 6107–6110. 12955090 10.1038/sj.onc.1206732 PMC4209839

[10] Huang L, Chen D, Liu D, Yin L, Kharbanda S, Kufe D (2005) MUC1 oncoprotein blocks glycogen synthase kinase 3beta-mediated phosphorylation and degradation of beta-catenin. Cancer Res 65: 10413–10422. 16288032 10.1158/0008-5472.CAN-05-2474

[11] Li Q, Ren J, Kufe D (2004) Interaction of human MUC1 and beta-catenin is regulated by Lck and ZAP-70 in activated Jurkat T cells. Biochem Biophys Res Commun 315: 471–476. 14766232 10.1016/j.bbrc.2004.01.075

[12] Duraisamy S, Ramasamy S, Kharbanda S, Kufe D (2006) Distinct evolution of the human carcinoma-associated transmembrane mucins, MUC1, MUC4 AND MUC16. Gene 373: 28–34. 16500040 10.1016/j.gene.2005.12.021

[13] Dharma Rao T, Park KJ, Smith-Jones P, Iasonos A, Linkov I, Soslow RA, et al (2010) Novel monoclonal antibodies against the proximal (carboxy-terminal) portions of MUC16. Appl Immunohistochem Mol Morphol 18: 462–472. 10.1097/PAI.0b013e3181dbfcd2 20453816 PMC4388147

[14] Corrales RM, Galarreta D, Herreras JM, Saez V, Arranz I, Gonzalez MJ, et al (2009) Conjunctival mucin mRNA expression in contact lens wear. Optom Vis Sci 86: 1051–1058. 10.1097/OPX.0b013e3181b4f02e 19661836

[15] Govindarajan B, Gipson IK (2010) Membrane-tethered mucins have multiple functions on the ocular surface. Exp Eye Res 90: 655–663. 10.1016/j.exer.2010.02.014 20223235 PMC2893012

[16] Kaneko O, Gong L, Zhang J, Hansen JK, Hassan R, Lee B, et al (2009) A binding domain on mesothelin for CA125/MUC16. J Biol Chem 284: 3739–3749. 10.1074/jbc.M806776200 19075018 PMC2635045

[17] Comamala M, Pinard M, Theriault C, Matte I, Albert A, Boivin M, et al (2011) Downregulation of cell surface CA125/MUC16 induces epithelial-to-mesenchymal transition and restores EGFR signalling in NIH:OVCAR3 ovarian carcinoma cells. Br J Cancer 104: 989–999. 10.1038/bjc.2011.34 21326240 PMC3065274

[18] Theriault C, Pinard M, Comamala M, Migneault M, Beaudin J, Matte I, et al (2011) MUC16 (CA125) regulates epithelial ovarian cancer cell growth, tumorigenesis and metastasis. Gynecol Oncol 121: 434–443. 10.1016/j.ygyno.2011.02.020 21421261

[19] Zorn KK, Tian C, McGuire WP, Hoskins WJ, Markman M, Muggia FM, et al (2009) The prognostic value of pretreatment CA 125 in patients with advanced ovarian carcinoma: a Gynecologic Oncology Group study. Cancer 115: 1028–1035. 10.1002/cncr.24084 19156927 PMC2664510

[20] Cancer Genome Atlas Research Network (2011) Integrated genomic analyses of ovarian carcinoma. Nature 474: 609–615. 10.1038/nature10166 21720365 PMC3163504

[21] Cheon DJ, Wang Y, Deng JM, Lu Z, Xiao L, Chen CM, et al (2009) CA125/MUC16 is dispensable for mouse development and reproduction. PLoS One 4: e4675 10.1371/journal.pone.0004675 19262696 PMC2650410

[22] Mazzoletti M, Broggini M (2010) PI3K/AKT/mTOR inhibitors in ovarian cancer. Curr Med Chem 17: 4433–4447. 21062259 10.2174/092986710794182999

[23] Ventura AP, Radhakrishnan S, Green A, Rajaram SK, Allen AN, O'Briant K, et al (2010) Activation of the MEK-S6 pathway in high-grade ovarian cancers. Appl Immunohistochem Mol Morphol 18: 499–508. 10.1097/PAI.0b013e3181e53e1c 20661131 PMC2989426

[24] Ghosh S, Basu M, Roy SS (2012) ETS-1 protein regulates vascular endothelial growth factor-induced matrix metalloproteinase-9 and matrix metalloproteinase-13 expression in human ovarian carcinoma cell line SKOV-3. J Biol Chem 287: 15001–15015. 10.1074/jbc.M111.284034 22270366 PMC3340257

[25] Strausberg RL, Feingold EA, Grouse LH, Derge JG, Klausner RD, Collins FS, et al (2002) Generation and initial analysis of more than 15,000 full-length human and mouse cDNA sequences. Proc Natl Acad Sci U S A 99: 16899–16903. 12477932 10.1073/pnas.242603899 PMC139241

[26] Xing D, Orsulic S (2006) A mouse model for the molecular characterization of brca1-associated ovarian carcinoma. Cancer Res 66: 8949–8953. 16982732 10.1158/0008-5472.CAN-06-1495 PMC1802660

[27] Lloyd KO, Yin BW (2001) Synthesis and secretion of the ovarian cancer antigen CA 125 by the human cancer cell line NIH:OVCAR-3. Tumour Biol 22: 77–82. 11125279 10.1159/000050600

[28] Seelenmeyer C, Wegehingel S, Lechner J, Nickel W (2003) The cancer antigen CA125 represents a novel counter receptor for galectin-1. JJ Cell Sci 116: 1305–1318. 12615972 10.1242/jcs.00312

[29] Liu Y, Zeng B, Zhang Z, Zhang Y, Yang R (2008) B7-H1 on myeloid-derived suppressor cells in immune suppression by a mouse model of ovarian cancer. Clin Immunol 129: 471–481. 10.1016/j.clim.2008.07.030 18790673

[30] Giannakouros P, Matte I, Rancourt C, Piche A (2015) Transformation of NIH3T3 mouse fibroblast cells by MUC16 mucin (CA125) is driven by its cytoplasmic tail. Int J Oncol 46: 91–98. 10.3892/ijo.2014.2707 25338620

[31] Xing D, Orsulic S (2005) A genetically defined mouse ovarian carcinoma model for the molecular characterization of pathway-targeted therapy and tumor resistance. Proc Natl Acad Sci U S A 102: 6936–6941. 15860581 10.1073/pnas.0502256102 PMC1087513

[32] Rao TD, Rosales N, Spriggs DR (2011) Dual-fluorescence isogenic high-content screening for MUC16/CA125 selective agents. Mol Cancer Ther 10: 1939–1948. 10.1158/1535-7163.MCT-11-0228 21817115 PMC3191303

[33] Shinoda Y, Ogata N, Higashikawa A, Manabe I, Shindo T, Yamada T, et al (2008) Kruppel-like factor 5 causes cartilage degradation through transactivation of matrix metalloproteinase 9. J Biol Chem 283: 24682–24689. 10.1074/jbc.M709857200 18617520 PMC3259811

[34] Taylor BS, Schultz N, Hieronymus H, Gopalan A, Xiao Y, Carver BS, et al (2010) Integrative genomic profiling of human prostate cancer. Cancer Cell 18: 11–22. 10.1016/j.ccr.2010.05.026 20579941 PMC3198787

[35] Heller G, Simonoff JS (1992) Prediction in censored survival data: a comparison of the proportional hazards and linear regression models. Biometrics 48: 101–115. 1581480

