# Supplementary material for: Expression of the Carboxy-Terminal Portion of MUC16/CA125 Induces Transformation and Tumor Invasion
Source: PLoS One. 2015 May 12;10(5):e0126633. doi: 10.1371/journal.pone.0126633 (PMC4429113; doi:10.1371/journal.pone.0126633)
Supplement: S1 ARRIVE Checklist — (PDF) [file pone.0126633.s001.pdf]

The ARRIVE Guidelines Checklist

Animal Research: Reporting In Vivo Experiments

Carol Kilkenny[1], William J Browne[2], Innes C Cuthill[3], Michael Emerson[4] and Douglas G Altman[5]

[1]The National Centre for the Replacement, Refinement and Reduction of Animals in Research, London, UK, [2]School of Veterinary Science, University of Bristol, Bristol, UK, [3]School of Biological Sciences, University of Bristol, Bristol, UK, [4]National Heart and Lung Institute, Imperial College London, UK, [5]Centre for Statistics in Medicine, University of Oxford, Oxford, UK.

	ITEM	RECOMMENDATION	Section/ Paragraph
Title	1	Provide as accurate and concise a description of the content of the article as possible.	Expression of the Carboxy-Terminal Portion of MUC16/ CA125 Induces Transformation and Tumor Invasion
Abstract	2	Provide an accurate summary of the background, research objectives, including details of the species or strain of animal used, key methods, principal findings and conclusions of the study.	The CA125 antigen is found in the serum of many patients with serous ovarian cancer and has been widely used as a disease marker. CA125 has been shown to be an independent factor for clinical outcome in this disease. In The Cancer Genome Atlas ovarian cancer project, MUC16 expression levels are frequently increased, and the highest levels of MUC16 expression are linked to a significantly worse survival. To examine the biologic effect of the proximal portion of MUC16/CA125, NIH/3T3 (3T3) fibroblast cell lines were stably transfected with the carboxy elements of MUC16. As few as 114 amino acids from the carboxy-terminus of MUC16 were sufficient to increase soft agar growth, promote matrigel invasion, and increase the rate of tumor growth in athymic nude mice. Transformation with carboxy elements of MUC16 was associated with activation of the AKT and ERK pathways. MUC16 transformation was associated with up-regulation of a number of metastases and invasion gene transcripts, including IL-1β, MMP2, and MMP9. All observed oncogenic changes were exclusively dependent on the extracellular "ectodomain" of MUC16. The biologic impact of MUC16 was also explored through the creation of a transgenic mouse model expressing 354 amino acids of carboxy-terminus of MUC16 ($MUC16^{c354}$). Under a CMV early enhancer plus chicken β actin promoter (CAG) $MUC16^{c354}$ was well expressed in many organs including the brain, colon, heart, kidney, liver, lung, ovary and spleen. $MUC16^{c354}$ transgenic animals appear to be viable, fertile, and have a normal lifespan. However, when crossed with p53-deficient mice, the $MUC16^{c354}$:$p53^{+/-}$ progeny displayed a higher frequency of spontaneous tumor development compared to $p53^{+/-}$ mice alone. We conclude that the carboxy-terminal portion of the MUC16/CA125 protein is oncogenic in NIH/3T3 cells, increases invasive tumor properties, activates the AKT and ERK pathways, and contributes to the biologic properties of ovarian cancer.
INTRODUCTION			

Background	3	a. Include sufficient scientific background (including relevant references to previous work) to understand the motivation and context for the study, and explain the experimental approach and rationale.<br>b. Explain how and why the animal species and model being used can address the scientific objectives and, where appropriate, the study's relevance to human biology.	The serum CA125 antigen has been a mainstay of ovarian cancer assessment and management since the early 1980's, but its biology and contribution to ovarian cancer manifestations have been poorly understood [1-3]. The cloning of CA125, achieved in 2001, first identified MUC16 as a tethered mucin with a small intracellular domain, a transmembrane domain, an ectodomain proximal to the putative cleavage site, and a large, heavily glycosylated region of 12-20 tandem repeats, each 156 amino acids long (Fig. 1A) [4-6]. Serous cancers of the ovary, fallopian tube, and uterus often express large amounts of MUC16, and aberrant MUC16 expression can be found in several other malignancies, including cancers of the lung, pancreas, and breast. Expression of other tethered mucins is a common feature of epithelial organs, and they are often over-expressed in malignancy. Two prominent examples are MUC1, which is over-expressed in many breast and ovarian cancers, and MUC4, which is characteristically abundant in pancreatic and gastrointestinal cancers [7]. Both of these mucins have been identified as having transforming properties [8, 9]. The transforming mechanisms are different and incompletely understood. MUC1 has a beta-catenin homology region that has been shown to translocate to the nucleus and act as a transcription factor [10, 11]. In contrast, MUC4 has HER-binding domains within its transmembrane region and acts, at least in part, through the HER family kinases [12, 13]. MUC16 lacks homologous regions to either of these domains and appears to have evolved independently [12]. Compared to both MUC1 and MUC4, the expression of MUC16 is more restricted and is normally confined, almost exclusively, to the Müllerian tract and the ocular epithelium [14-16]. The tandem repeat regions of the MUC16 molecule appear to function as key interacting proteins with mesothelin and other stromal proteins [17]. These interactions are probably responsible for the classic patterns of serosal spread by ovarian cancers. In clinical settings, high levels of the circulating elements from MUC16, which encode the CA125 antigen, are associated with an adverse clinical outcome, independent of stage, grade and other traditional clinical factors [18]. Amplification of genomic regions encoding MUC16 in ovarian cancer DNA and over-expression of MUC16 mRNA have been observed in The Cancer Genome Atlas (TCGA) ovarian cancer project and is associated with worse outcome [19]. Loss of MUC16 in the mouse is not associated with a distinct phenotype, but the effect of persistent or aberrant MUC16 expression is not known [20]. We hypothesized MUC16 is important in the serous ovarian cancer phenotype and suspected that expression of key elements from the MUC16 protein could promote invasive behavior in preclinical models of cancer, both *in vitro* or *in vivo*. Our experimental results strongly indicate that the expression of the most proximal MUC16 fragments is associated with specific alterations of signal transduction, gene expression, and aggressive biological behavior.

Objectives	4	Clearly describe the primary and any secondary objectives of the study, or specific hypotheses being tested.	The CA125 antigen has been linked with ovarian cancer for more than 20 years, and serum CA125 is a known prognostic factor in the management of this disease. This manuscript is the first to show that even truncated versions of the MUC16 gene induce transformation in 3T3 fibroblasts. We also demonstrate that MUC16 expression increases the *in vivo* aggressiveness of ovarian cancer cell lines through increased expression of key invasiveness genes. Increased phosphorylation of both AKT and ERK is also linked to the MUC16 expression. This expression of MUC16 also increases growth in nude mouse models. These effects are seen in ovarian cancer cell lines as well. We show that this effect is dependent on N-glycosylation of the proximal portion of MUC16. These studies are confirmed with the first description of a newly developed transgenic MUC16 mouse which develops spontaneous tumors when crossed with p53+/- mice. We also show that analysis of The Cancer Genome Atlas data set confirms the adverse impact of high MUC16 expression serious ovarian cancer. These findings will support future drug development directed at this novel, tumor-specific target.
METHODS			
Ethical statement	5	Indicate the nature of the ethical review permissions, relevant licences (e.g. Animal [Scientific Procedures] Act 1986), and national or institutional guidelines for the care and use of animals, that cover the research.	Transfected cell lines and appropriate control cell lines were introduced into the flank or peritoneal cavity of athymic nude mice, and routine animal care was provided by the MSKCC Antitumor Assessment Core Facility. The MSKCC Mouse Genetics Core Facility performed the microinjection procedure on B6CBAF1/J mice. All experimental animals were maintained in accordance with the guidelines approved by the MSKCC Institutional Animal Care and Use Committee and Research Animal Resource Center and the NIH Guide for the Care and Use of Laboratory Animals. All work described in this manuscript was described and performed under the MSKCC Animal Welfare Protocol 09-03-003, which is reviewed and approved annually.
Study design the	6	For each experiment, give brief details of study design including:<br>a. The number of experimental and control groups.<br>b. Any steps taken to minimise the effects of subjective bias when allocating animals to treatment (e.g. randomisation procedure) and when assessing results (e.g. if done, describe who was blinded and when).<br>c. The experimental unit (e.g. a single animal, group or cage of animals).<br>A time-line diagram or flow chart can be useful to illustrate how complex study designs were carried out.	a. As shown in our Figs. 2, 3, 4 and 5 and 'Tumor Growth in Athymic Nude mice' in the 'Materials And Methods section'. Two or three experimental groups were always compared with the control phrGFP group within the selected cell line.<br>b. Randomised procedures were undertaken and nothing was blinded in assessing the results.<br>c. 5 mice/cage per group was maintained for the study
Experimental experimental	7	For each experiment and each group, including controls, procedures provide precise details of all procedures carried out. For example:<br>a. How (e.g. drug formulation and dose, site and route of administration, anaesthesia and analgesia used [including monitoring], surgical procedure, method of euthanasia). Provide details of any specialist equipment used, including supplier(s).<br>b. When (e.g. time of day).<br>c. Where (e.g. home cage, laboratory, water maze).<br>d. Why (e.g. rationale for choice of specific anaesthetic, route of administration, drug dose used).	a. No drugs, No ansesthesia, No analgesia, No painful procedure or any special equipment were used in this study. Just cell line s.c. implantation with daily monitoring of mice were used in this study.<br>b. 10 AM – monitoring of mice<br>c. Animal Room<br>d. No drugs or anaesthesia were used in this study.

Experimental<br><br>median<br><br>mean	8	a. Provide details of the animals used, including species, strain, sex, animals developmental stage (e.g. mean or age plus age range) and weight (e.g. or median weight plus weight range).<br>b. Provide further relevant information such as the source of animals, international strain nomenclature, genetic modification status (e.g. knock- out or transgenic), genotype, health/ immune status, drug or test naïve, previous procedures, etc.	a. Mice, Athymic nu/nu, Female, 6-8 week old Adult mice weighing around 20 gms.<br><br>b. Nude mice (*nu/nu*; Harlan Laboratories) were used for *in vivo* studies and were cared for in accordance with guidelines approved by the Memorial Sloan-Kettering Cancer Center Institutional Animal Care and Use Committee (Protocol # 09-03-003) and Research Animal Resource Center. Naïve immuno compromised mice which were never been used for any other drug or test procedures were used for this study.<br><br>For transgenic model mice were described in 'Materials and Methods' under 'MUC16^{c354} transgenic mice' section.

Housing and husbandry	9	Provide details of:<br>a. Housing (type of facility e.g. specific pathogen free [SPF]; type of cage or housing; bedding material; number of cage companions; tank shape and material etc. for fish).<br>b. Husbandry conditions (e.g. breeding programme, light/dark cycle, temperature, quality of water etc for fish, type of food, access to food and water, environmental enrichment).<br>c. Welfare-related assessments and interventions that were carried out prior to, during, or after the experiment.	a. Upon arrival at MSKCC Animal Room facility, the mice were kept under observation for a minimum of seven days in specific pathogen free (SPF) type cage. In all instances there were no significant findings indicative of poor health and therefore all of the mice were deemed suitable for study.<br><br>b. Each mouse was allowed ad libitum access to Certified Rodent Diet # 5053 (LabDiet) and acidified reverse-osmosis water during the entire study period. The mice were housed in individually ventilated polysulfone cages (Thoren) on aspen chip bedding (PWI Industries) and kept in an animal room that was maintained between 72.0-76.0^{O}F with a relative humidity of 39-76% during the study. Room lights were controlled by an automatic timer set to provide 12 hours of light (6am-6pm) and 12 hours of night per day. Cage size and animal care conformed to the guidelines of the Guide for the Care and Use of Laboratory Animals (7th edition; Institute of Laboratory Animal Resources, Commission of Life Sciences, National Research Council, National Academy Press; Washington, D.C., 1996).<br><br>c. Daily monitoring mice for welfare related assessments.
Sample size	10	a. Specify the total number of animals used in each experiment, and the number of animals in each experimental group.<br>b. Explain how the number of animals was arrived at. Provide details of any sample size calculation used.<br>c. Indicate the number of independent replications of each experiment, if relevant.	a. Details were given under the Figure legend section for each experiment. A total of 45 mice for 3 group experiment or a total of 80 mice for 4 group experiments were undertaken for this study. 15 – 20 mice per group were used in each experiment.<br><br>b. The number of animals were arrived after an intial pilot experiment of 5 mice per group.
Allocating animals to experimental groups	11	a. Give full details of how animals were allocated to experimental groups,including randomisation or matching if done.<br>b. Describe the order in which the animals in the differentexperimental groups were treated and assessed.	a. Mice were allocated to groups by randomization.<br><br>b. Not applicable
Experimental outcomes outcomes	12	Clearly define the primary and secondary experimental assessed (e.g. cell death, molecular markers, behavioural changes).	Tumor volume was measured by calipers twice weekly.<br><br>For transgenic mice in Figure legends section for Fig. 6D and S4 Fig., H&E staining was performed.

Statistical methods	13	a. Provide details of the statistical methods used for each analysis.<br>b. Specify the unit of analysis for each dataset (e.g. single animal, group of animals, single neuron).<br>c. Describe any methods used to assess whether the data met the assumptions of the statistical approach.	a. Details are given in the 'Materials and Methods' under 'Statistical Analysis' section.<br>b. The comparisons of the tumor volumes were made using area under the curve assessments for total tumor volume over time in each animal. The assessment of tumor volume was made based on the last day that all animals were alive in both groups. A non-parametric test for ranks (Wilcoxon two sample test) was used to test for a difference in distributions among the groups. In the animal survival studies, a time to event analysis was performed, with the event defined as time to tumor volume exceeding 1500 mm^3. Animals with tumor volume less than 1500 mm^3 were followed for up to 60 days and then censored. The Kaplan-Meier method was used to estimate survival distribution [32].<br>c. Not Applicable
RESULTS			
Baseline data	14	For each experimental group, report relevant characteristics and health status of animals (e.g. weight, microbiological status, and drug or test naïve) prior to treatment or testing. (This information can often be tabulated).	Healthy, average weight 20 gms and drug naive
Numbers analysed	15	a. Report the number of animals in each group included in each analysis. Report absolute numbers (e.g. 10/20, not 50%[2]).<br>b. If any animals or data were not included in the analysis, explain why.	a. All mice in each group for this study were included in the analysis.<br>b. Not Applicable
Outcomes and estimation	16	Report the results for each analysis carried out, with a measure of precision (e.g. standard error or confidence interval).	Mean $\pm$ Standard Error were reported in the study
Adverse events	17	a. Give details of all important adverse events in each experimental group. b. Describe any modifications to the experimental protocols made to reduce adverse events.	a.None.<br>b. None
DISCUSSION			

Interpretation/ scientific implications	18	a. Interpret the results, taking into account the study objectives and hypotheses, current theory and other relevant studies in the literature.<br>b. Comment on the study limitations including any potential sources of bias, any limitations of the animal model, and the imprecision associated with the results[2].<br>c. Describe any implications of your experimental methods or findings for the replacement, refinement or reduction (the 3Rs) of the use of animals in research.	This is covered under the 'Discussion' section paragraph 1, 2, 3 and 4.<br><br>a.MUC16, encoding the CA125 antigen, circulates in the plasma of many patients with ovarian cancer [1]. MUC16 is unique among the tethered mucins for its limited expression outside mullerian tissues [2, 14]. While increasingly viewed as an adverse prognostic factor independent of tumor bulk, the biologic mechanism for its negative impact has not been well understood [18]. The NH_2-portion of the molecule contains multiple tandem repeats that encode the CA125 antigen and appear to serve as important adhesion partners to mesothelin and some galectins (Fig. 1A) [5, 17, 24-26]. While these adhesion functions have been suggested to be critical in MUC16-related adverse outcome, these studies do not explain all of the observed changes in ovarian cancer cell behavior. The cloning of the MUC16 glycoprotein has provided basic structural information about the MUC16 gene product [4, 5]. These observations are the first data to indicate that MUC16 may mediate signaling from the environment into the cancer cell. In these studies, we have demonstrated that as little as 114 amino acids from the carboxy-terminus of MUC16 are sufficient to transform NIH/3T3 (3T3) cells, supporting both increased soft agar growth and increased matrigel invasiveness. These changes are associated with an altered gene-expression profile and increased expression of critical invasion genes such as MMP2, MMP9, CXCL12, and CDH11. While longer elements induce a more virulent behavior, even the residual 114 amino acids proximal to the putative cleavage site are sufficient in 3T3 cells to induce some of the same changes in invasion gene expression. These findings are most consistent with an "outside in" signal transduction by the most proximal portions of the protein, including a residual extracellular domain along with the transmembrane domain and cytoplasmic tail. That signal appears to activate a transcription of a gene program that facilitates the implantation and growth of MUC16 expressing cells in soft agar and nude mice. When the transfected cells are examined for activation of common oncogenic pathways, both AKT and ERK pathways appear to be activated by constitutive expression of MUC16. The mechanism by which MUC16 increases AKT/ERK phosphorylation is unclear and will require further studies. Although MUC16 sequences are very different, other tethered mucins, including both MUC1 and MUC4, have been shown to act as signal-generating oncogenes in 3T3 cells [9] and rat fibroblasts [8]. It is likely that the role of mucins on the cancer cell surface play important roles through mechanisms that are still being defined. Based on the findings in 3T3 cells, the results of the MUC16 transgenic mouse experiment is highly supportive. By itself, the same MUC16 proximal 354 sequence could be readily expressed in nearly all murine tissues with no adverse effect in the transgenic mouse. The rate of spontaneous tumor formation was very low in those mice, and reproductive function seemed unaffected. However, like other murine ovarian cancer models, loss of p53 function appears to play a strong permissive role in MUC16-dependent tumor formation [27]. These results certainly are consistent with uniform p53 inactivation, which characterizes ovarian cancer in the TCGA data set. These findings are the first to describe MUC16-linked changes in cellular behavior and gene transcription. The *in vitro* and *in vivo* models are consistent with the adverse effects of MUC16 expression levels in serous ovarian cancer and promote the understanding of MUC16 as a pathogenic contributor to the behaviors of ovarian cancer. The adverse impact of increasing CA125 expression is consistent with increased *in vivo* tumor growth and lethality of MUC16 (+) 3T3 transfectants [18]. Additional work of the precise mechanisms initiatingMUC16 signaling, the effects of MUC16 binding to stromal elements, and the identification of the key molecular partners of cell surface MUC16 are likely to further enhance our understanding of ovarian cancer biology.<br><br>a.

Generalisability/ translation	19	Comment on whether, and how, the findings of this study are likely to translate to other species or systems, including any relevance to human biology.	These findings are the first to describe MUC16-linked changes in cellular behavior and gene transcription. The *in vitro* and *in vivo* models are consistent with the adverse effects of MUC16 expression levels in serous ovarian cancer and promote the understanding of MUC16 as a pathogenic contributor to the behaviors of ovarian cancer. The adverse impact of increasing CA125 expression is consistent with increased *in vivo* tumor growth and lethality of MUC16 (+) 3T3 transfectants [18]. Additional work of the precise mechanisms initiatingMUC16 signaling, the effects of MUC16 binding to stromal elements, and the identification of the key molecular partners of cell surface MUC16 are likely to further enhance our understanding of ovarian cancer biology.
Funding	20	List all funding sources (including grant number) and the role of the funder(s) in the study.	This work supported by NIH Grant # PO1-CA52477-17. This work used core facilities funded by NIH Core Grant # P30 CA 008748. The NIH played no role in any aspect of study design, data collection and analysis, or publication.

References:

1. Kilkenny C, Browne WJ, Cuthill IC, Emerson M, Altman DG (2010) Improving Bioscience Research Reporting: The ARRIVE Guidelines for Reporting Animal Research. *PLoS Biol* 8(6): e1000412. doi:10.1371/journal.pbio.1000412
2. Schulz KF, Altman DG, Moher D, the CONSORT Group (2010) CONSORT 2010 Statement: updated guidelines for reporting parallel group randomised trials. *BMJ* 340:c332.
