# Supplementary material for: Expression of the Carboxy-Terminal Portion of MUC16/CA125 Induces Transformation and Tumor Invasion
Source: PLoS One. 2015 May 12;10(5):e0126633. doi: 10.1371/journal.pone.0126633 (PMC4429113; doi:10.1371/journal.pone.0126633)
Supplement: S1 Data — (DOC) [file pone.0126633.s002.doc]

**S1 Data**: These data contain supporting Materials and Methods information, including a summarization of the characteristics of the MUC16 transfectants.

Forward and Reverse primers for MUC16-cytoplasmic Domain (MUC16^c114^, **carboxy-terminus 114 aa**) (5’ **CCA TGC GAT ATC *GCC ACC ATG G*TG AAC TTC TCG CCA CTG GCT** 3’ and 5’ **TAC GGC GGC CGC TTG CAG ATC CTC CAG GTC TAG G** 3’). MUC16^c344^ (one tandem repeat which has only one cysteine loop, 344 aa) (5’ **CCA TGC GAT ATC *GCC ACC ATG G*TG ACA GGC CCT GGG CTG GAC AGA**  3’ and 5’ **TAC GGC GGC CGC TTG CAG ATC CTC CAG GTC TAG G** 3’) with EcoRV and KOZAK in the forward primer and NotI in the reverse primer. MUC16^c114^–GFP DNA construct was used to create MUC16^c80^ and MUC16^c86^ constructs by quick change primers. Forward and Reverse primers for MUC16^c57-114^ pFUSE-hIgG1-Fc2 vector (**5' C CAT GCG ATA TCA AAC TTC TCG CCA CTG GCT 3’** and **5’ AGA TCT AAC CAT GGG AAG GTC AGA ATT CCC AGT 3’)** and Forward and Reverse primers for the sugar binding domain of ^117-244^LGALS3 for pFUSE-hIgG1-Fc2 vector **(5’ C CAT GC**G ATA TCA CCT TAT AAC CTG CCT TTG 3’and 5’ AGA TCT AAC CAT **GGT ATA TGA AGC ACT GGT** 3’) with EcoRV in the forward primer and NcoI in the reverse primer. All the above primers were synthesized by Sigma Genosys, The Woodlands, TX.

PCR conditions were achieved by the following processes: DNA was melted at 95°C for 5 minutes in order to achieve denaturation. Thirty repeat cycles of heating at 97°C for 30 seconds, annealing at 60°C for 1 minute, and extending at 72°C for 1 minute were conducted. This was followed by an extension of the generated PCR product strand at 72°C for 5 minute and then cooled at 4°C overnight.

phrGFP II-C vector DNA, MUC16^c114^, MUC16^c80^, MUC16^c86^ and MUC16^c344^ gel purified DNAs individually digested overnight at 37^o^C water bath with EcoRV and NotI (New England Biolabs, Beverly, MA) restriction enzymes. pFUSE-hIgG1-Fc2 vector DNA, MUC16 ^c57-114^ and ^117-244^LGALS3 gel purified DNAs individually digested overnight at 37^o^C water bath with EcoRV and NcoI (New England Biolabs, Beverly, MA) restriction enzymes. MUC16^c114^ and phrGFP; MUC16^c344^ and phrGFP; restrict digested DNA’s were gel purified and ligated overnight using T4 Ligase (Roche Diagnostics Corporation, Indianapolis, IN). Similarly, MUC16 ^c57-114^ and pFUSE-hIgG1-Fc2; ^117-244^LGALS3 and pFUSE-hIgG1-Fc2 restrict digested DNA’s were gel purified and ligated overnight using T4 Ligase (Roche Diagnostics Corporation, Indianapolis, IN). Ligated DNA were transformed into XL-1 Blue super competent cells (Stratagene, La Jolla, CA) following manufacturer’s protocol and plated them on agar plates with LB medium containing Kanamycin (50 μg/mL, Sigma Chemical Co., St. Louis, MO) for phrGFP vectors or on agar plates with LB medium containing 25 ug/ml of Zeocin (Invitrogen, CA). Clones were selected the following day and Miniprep DNA was extracted using Wizard Plus Miniprep DNA purification system (Promega Corporation, Madison, WI). Selected clones DNA was sequence at MSKCC DNA sequencing core facility using forward and reverse primers of MUC16 and phrGFP to confirm MUC16 and phrGFP presence in the sequences as a fused constructs or MUC16^c57-114^ and pFUSE-hIgG1-Fc2 or ^117-244^LGALS3 and pFUSE-hIgG1-Fc2. Megaprep DNA from such clones were made by using Wizard Plus Megaprep DNA purification system (Promega Corporation, Madison, WI) which were also confirmed for the presence of MUC16 and phrGFP or MUC16^c57-114^ and pFUSE-hIgG1-Fc2 or ^117-244^LGALS3 and pFUSE-hIgG1-Fc2 in their sequences as a fused constructs.

**FACS Analyses**

Transfected cells were trypsinized, washed and counted by haemocytometer. Cells were distributed into multiple eppendorf tubes with at least 0.5-1 X 10^6^/tube. Cells were washed with PBS containing 1% FCS and 0.025% Sodium Azide (FACS buffer). For surface FACS staining, cells were incubated either without (for second antibody control) or with 1 μg/tube of bioreactive supernatants of MUC16-carboxy-terminus monoclonals (4H11.2.5), Mouse anti-human OC125 (M3519) (DakoCytomation, Dako North America Inc., Carpinteria, CA) for 30 minutes on ice. Cells in eppendorf tubes were also for surface stained with 1 μg/tube of non-specific isotype matched control mouse antibodies (13C4 for IgG1 and 4E11 for IgG2b monoclonals obtained from MSKCC Monoclonal core facility)(data not shown) and incubated on ice for 30 minutes. All cells were washed 3 times with FACS buffer. Cells were incubated with 1 μg/tube of second antibody Goat anti-mouse IgG1-PE or IgG2b-PE for 30 minutes on ice and then washed 3 times with FACS buffer. The cells were analyzed by FACS Calibur machine at MSKCC Flow Cytometry Core Facility. Geometric Mean 4H11-PE Fluorescence data were presented in S2 Fig.
