# Supplementary figures and images for: Expression of the Carboxy-Terminal Portion of MUC16/CA125 Induces Transformation and Tumor Invasion
Source: PLoS One. 2015 May 12;10(5):e0126633. doi: 10.1371/journal.pone.0126633 (PMC4429113; doi:10.1371/journal.pone.0126633)

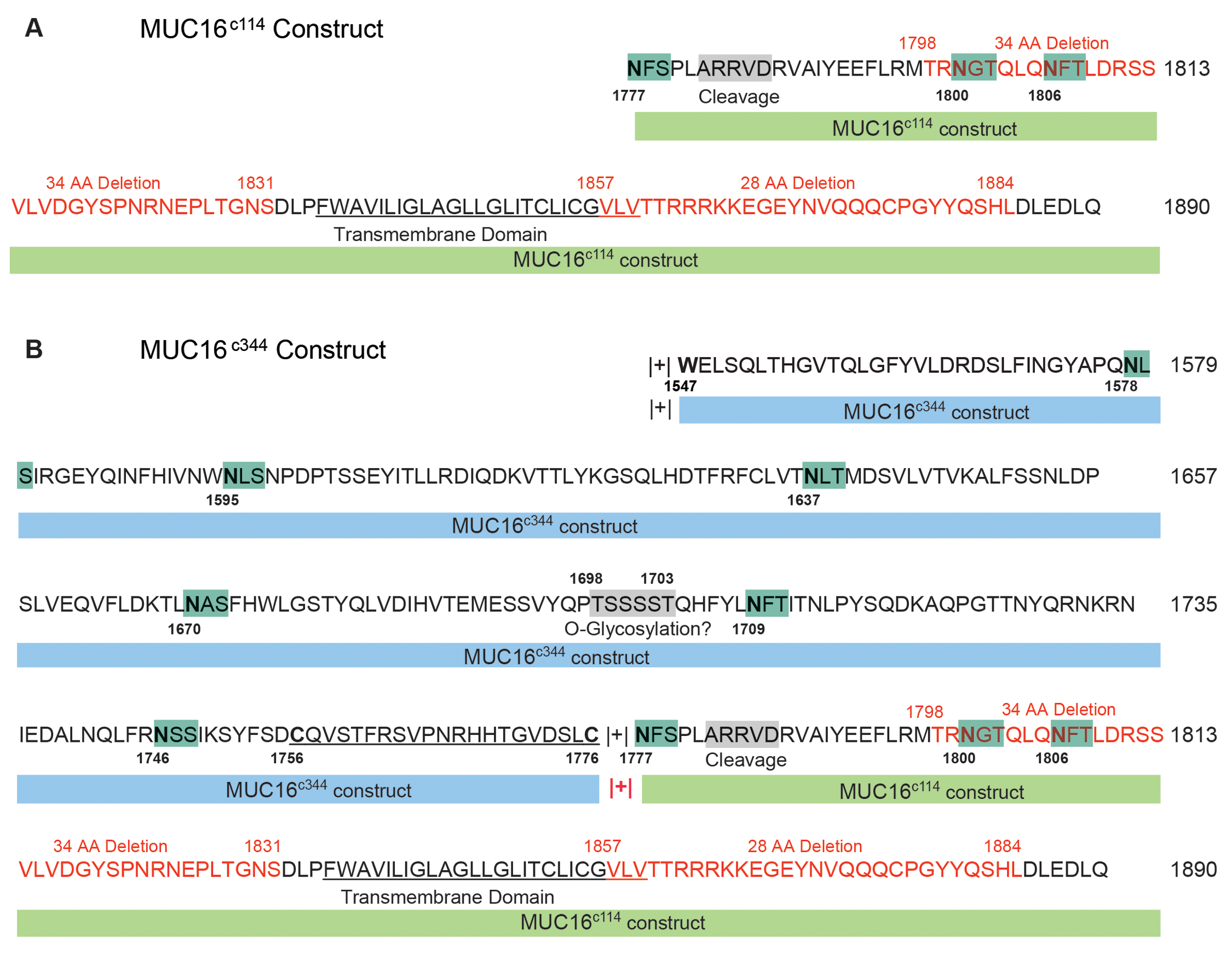

Supplement: S1 Fig — The N-glycosylation sites are highlighted in dark green; the O-glycosylation site is highlighted in gray; and the transmembrane domain (1835 to 1859) is underlined. The 28 amino acid internal domain deletion (1857–1884) and the 34 amino acid ectodomain deletion (1798–1831) are noted in red letters. (TIF) [file pone.0126633.s003.tif]

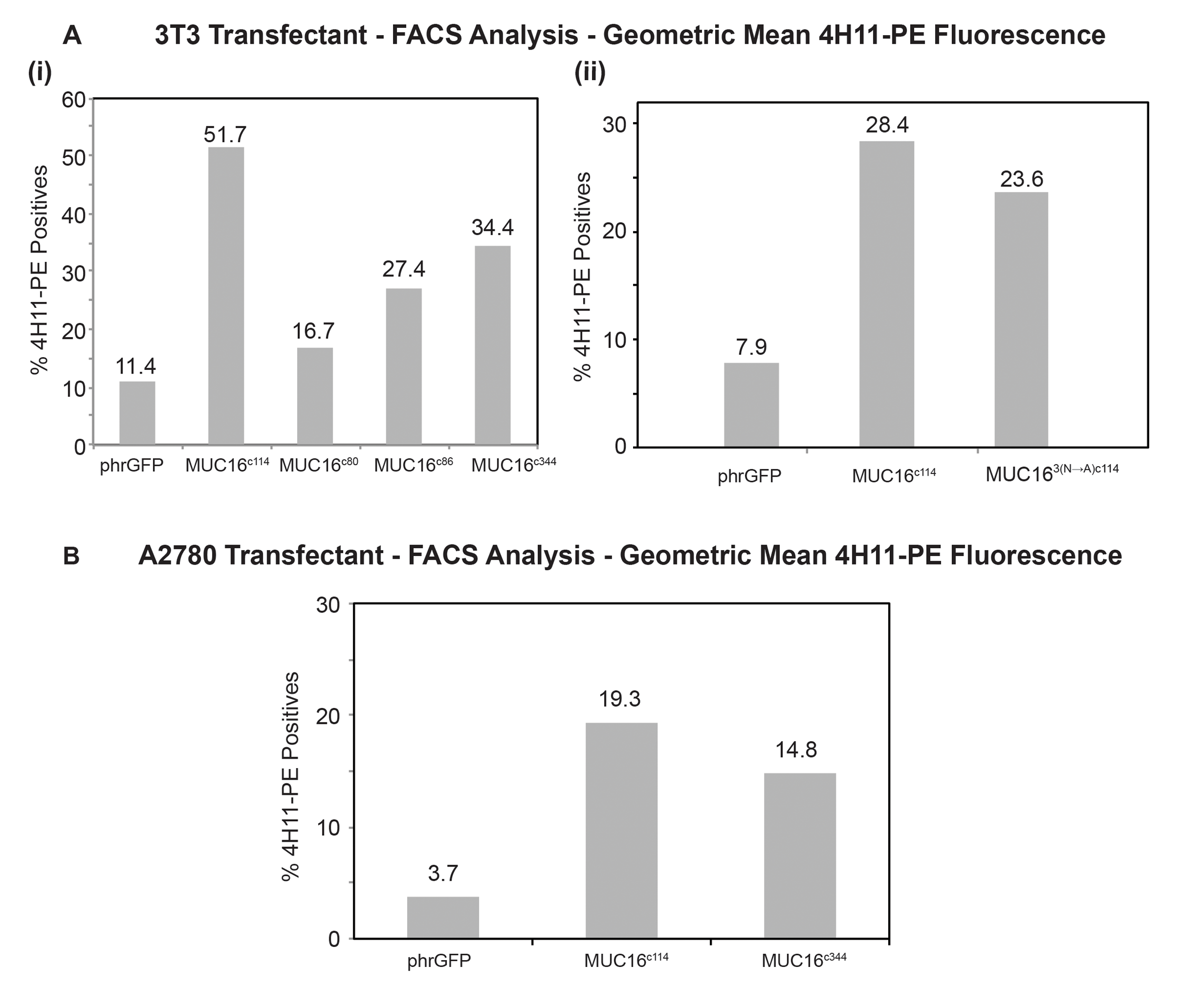

Supplement: S2 Fig — In each case, substantial MUC16 is present on the cell surface. (TIF) [file pone.0126633.s004.tif]

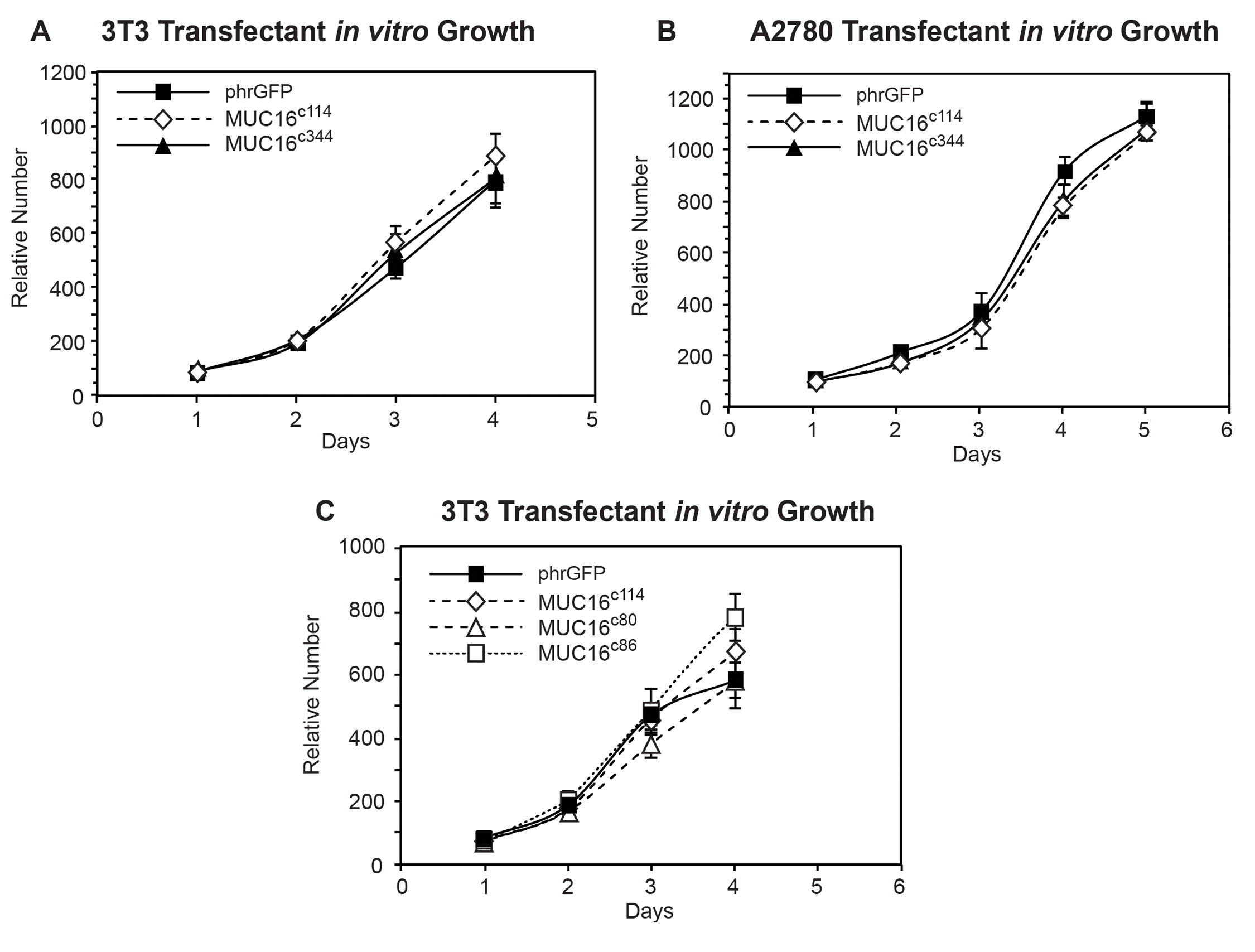

Supplement: S3 Fig — Panels A and B include a GFP vector control, a MUC16c114-GFP minimal carboxy element, and a more extended expression vector. In each case, the growth is supported by 10% heat inactivated calf serum. The growth of 3T3 cells was reduced for all cell lines in media with 1% heat inactivated calf serum. The MUC16c344-GFP was introduced into 3T3 and A2780 cell lines. Panel C describes the in vitro growth of the deletion mutants. No statistical differences are seen among any of the curves. (TIF) [file pone.0126633.s005.tif]

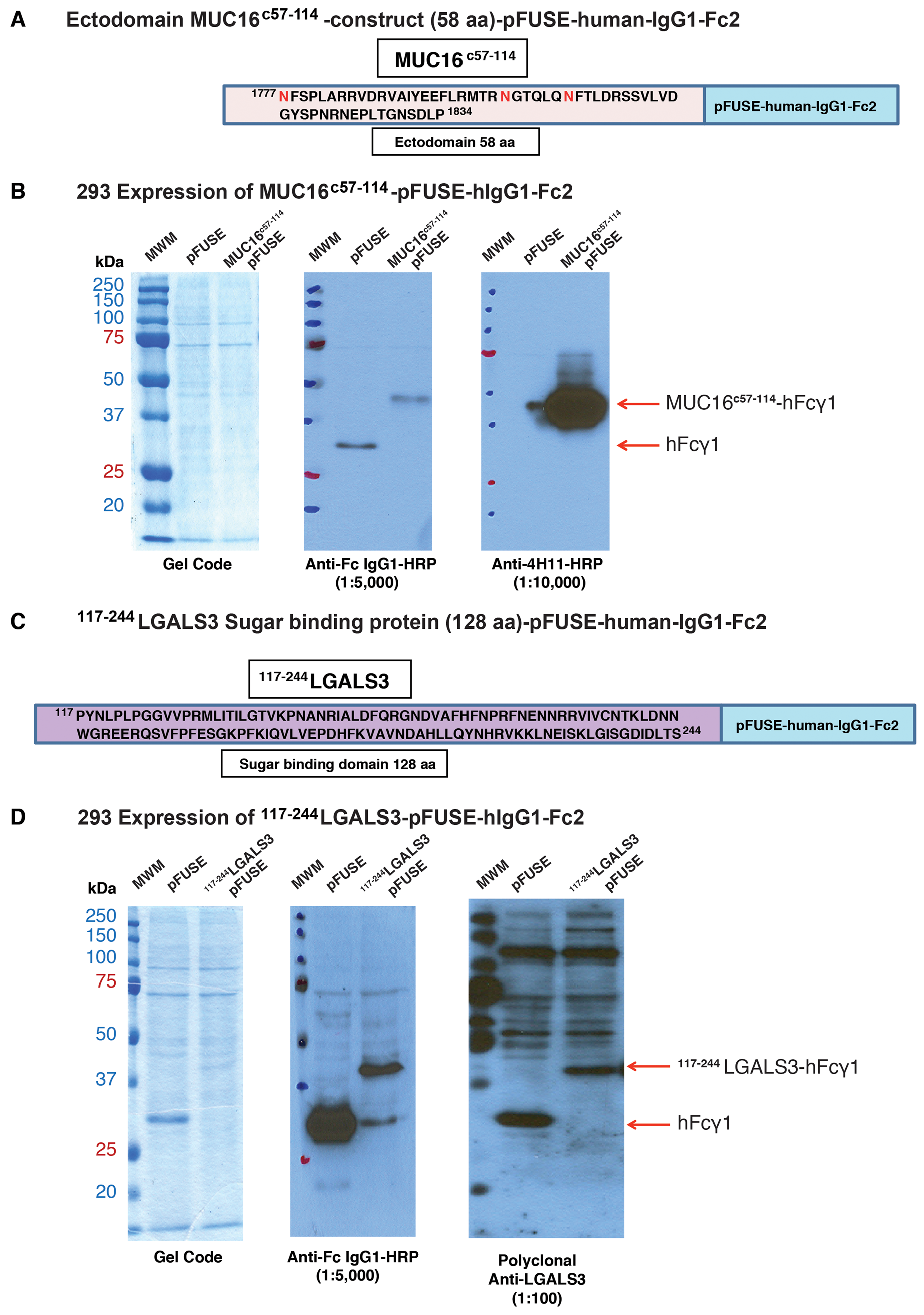

Supplement: S4 Fig — S4B Fig shows 293 cell expression of MUC16c57-114pFUSE-hIgG1-Fc2 fusion protein, Western blot. S4C Fig shows the 117-244LGALS3 amino acid sequence inserted into pFUSE-hIgG1-Fc2, resulting in the 117-244LGALS3pFUSE-hIgG1-Fc2 vector. S4D Fig shows 293 cell expression of 117-244LGALS3pFUSE-hIgG1-Fc2 fusion protein, Western blot. (TIF) [file pone.0126633.s006.tif]

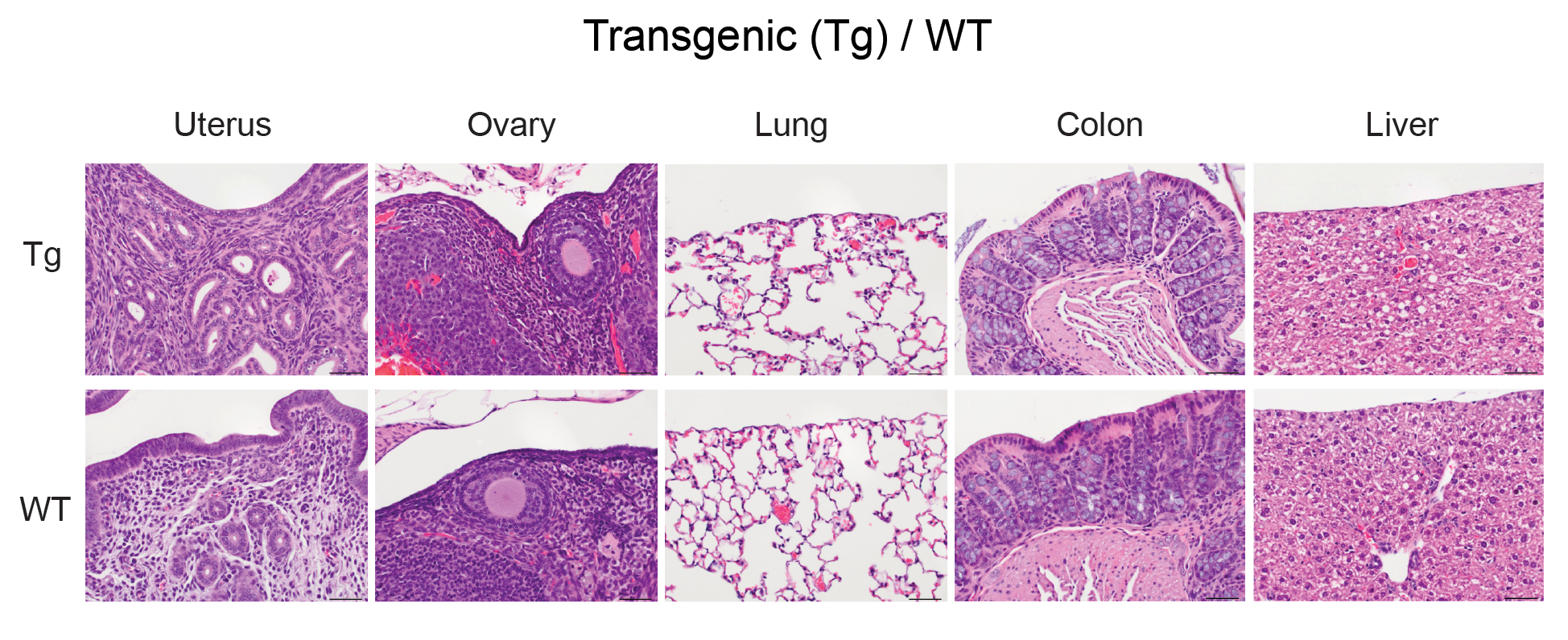

Supplement: S5 Fig — Tissue sections were stained with hematoxylin and eosin (scale bar: 50 μm). Uterine endometrial hyperplasia was observed with similar incidence and severity in both genotypes (here only shown in the transgenic animal). The ovary, lung, colon and liver of transgenic animals (Tg) were similar to the parental line (wild type, WT). (TIF) [file pone.0126633.s007.tif]
